# The small-secreted cysteine-rich protein CyrA is a virulence factor participating in the attack of *Caenorhabditis elegans* by *Duddingtonia flagrans*

**DOI:** 10.1371/journal.ppat.1010028

**Published:** 2021-11-04

**Authors:** Nicole Wernet, Valentin Wernet, Reinhard Fischer

**Affiliations:** Karlsruhe Institute of Technology (KIT)—South Campus, Institute for Applied Biosciences, Dept. of Microbiology, Karlsruhe, Germany; Chinese Academy of Sciences, CHINA

## Abstract

Nematode-trapping fungi (NTF) are a diverse and intriguing group of fungi that live saprotrophically but can switch to a predatory lifestyle when starving and in the presence of nematodes. NTF like *Arthrobotrys oligospora* or *Duddingtonia flagrans* produce adhesive trapping networks to catch and immobilize nematodes. After penetration of the cuticle, hyphae grow and develop inside the worm and secrete large amounts of hydrolytic enzymes for digestion. In many microbial pathogenic interactions small-secreted proteins (SSPs) are used to manipulate the host. The genome of *D*. *flagrans* encodes more than 100 of such putative SSPs one of which is the cysteine-rich protein CyrA. We have chosen this gene for further analysis because it is only found in NTF and appeared to be upregulated during the interaction. We show that the *cyrA* gene was transcriptionally induced in trap cells, and the protein accumulated at the inner rim of the hyphal ring before *Caenorhabditis elegans* capture. After worm penetration, the protein appeared at the fungal infection bulb, where it is likely to be secreted with the help of the exocyst complex. A *cyrA*-deletion strain was less virulent, and the time from worm capture to paralysis was extended. Heterologous expression of CyrA in *C*. *elegans* reduced its lifespan. CyrA accumulated in *C*. *elegans* in coelomocytes where the protein possibly is inactivated. This is the first example that SSPs may be important in predatory microbial interactions.

## Author summary

Pathogenic microorganisms are living at the expense of their host organisms and immediate killing of the host may be disadvantageous. Therefore, many bacterial or fungal pathogens developed an arsenal of small-secreted proteins during the colonization to modulate their host for instance to suppress its defense reactions. This allows a biotrophic phase, at least for some time. Some higher eukaryotic “pathogens” also live at the expense of their hosts but are often predators. In this case, quick killing is followed by digestion. In the case of predatory fungi, one could expect a similar situation, quick killing followed by digestion. However, the genome of such fungi encodes many putative small-secreted proteins, and we show here that one of them indeed appears to be secreted into the host and contributes to virulence. The protein is produced in the trapping devices of the fungus and especially in the penetration peg right after entering the nematode. Heterologous expression in *Caenorhabditis elegans* reduced the lifespan of the worms. This protein is to our knowledge the first characterized small-secreted protein in the predatory relationship between fungi and nematodes.

## Introduction

Nematode-trapping fungi (NTF) are carnivorous microorganisms that can trap and digest nematodes with sophisticated trapping structures. Most fungi in this versatile group can live saprotrophically and are able to switch to a predatory lifestyle while others are obligate pathogens [1]. A broad variety of diverse trapping structures is found in different species, such as adhesive networks, constricting- and non-constricting rings and adhesive knobs. NTF play an important role in the regulation of the nematode population in soil in almost all known ecosystems [1]. Further, as natural antagonists of nematodes they have powerful potential to be used as biocontrol agent against animal and plant pathogenic nematodes [2,3].

Nematodes cause agricultural losses of 80 billion US dollars worldwide [4]. This is a devastating number especially today, where we must face the needs of a growing population. The use of nematicides is limited because of their negative effect on the environment and health concerns [5,6]. Nematodes are important plant pathogens but can also be a problem in animals. Livestock, like sheep or cows infected with pathogenic nematodes have a reduced productivity and lifespan [7,8]. Nematode eggs are spread onto the pasture with the feces where they go through their life cycle and then are re-ingested during grazing leading to re-infection. This vicious cycle can be broken by feeding pellets containing NTF spores to the animals [2,9]. *Duddingtonia flagrans* is well suited for such an application because it produces robust and resistant chlamydospores besides adhesive, three-dimensional trapping networks [10–12]. It has already been established successfully as a biocontrol agent in horses, cattle and other animals [13,14]. The chlamydospores survive the passage through the gastrointestinal tract and are then able to germinate in the feces where they reduce the number of nematodes. The genome of this fungus has been sequenced and annotated recently, and molecular and cell biological methods were developed [15]. This opens new avenues for basic research but may also help to optimize the fungus as biocontrol agent.

Most NTF belong to the monophyletic group in the order Orbiliales (Ascomycota), and the co-evolution with their nematode prey dates back 400 million years. Their occurrence in many taxonomic groups is an indicator that they evolved independently serval times during evolution [16]. They are optimal model systems to study predator-prey interactions between eukaryotes and the underlying interspecies communication [17,18]. It is known that NTF induce trap formation in the presence of nematodes after sensing nematode-specific ascarosides [18,19]. On the other hand, the fungi lure the nematodes to the traps by secreting volatiles that are attractive to the worms [17,19]. After nematodes are trapped, hyphae penetrate the cuticle, secrete many hydrolytic enzymes and digest the worms [20]. An open question is if proteins with no obvious enzymatic activity are also required for the interaction or if the fungus quickly kills the worm and digests the dead material afterwards. In other pathogenic associations such proteins exist and since they are mostly rather small proteins and are secreted into the host, they were named small-secreted proteins (SSPs). They are also called virulence factors or effectors depending on the organismic interaction type and if there is a longer biotrophic phase, where effector proteins e.g. suppress the defense reactions of the host [21]. They are also crucial in microbial symbiotic interactions [22].

Secretion of the SSPs can follow the conventional secretion pathway or an alternative route, as it was shown in the rice-blast fungus *Magnaporthe oryzae*. Here, the cytoplasmic effector Pwl2 is secreted in a Golgi-independent manner, while the apoplastic effector Bas4 uses the conventional pathway [23]. Secretion of the cytoplasmic effector depends on the exocyst complex which is therefore essential for plant infection [23,24]. The complex is important for the correct spatiotemporal regulation of exocytosis and guides the docking and tethering of vesicles to the target membrane [25]. Another example of different secretion pathways for SSPs are *Phytophthora sojae* and *Verticillium dahlia* effectors that suppress salicylate-mediated innate immunity *in planta* [26].

Although the necessity of SSPs in predatory interactions is not obvious, many genes of NTF encode such small proteins, and many of the genes are transcriptionally upregulated during the nematode attack and are found in the pathogen-host interaction (PHI base) database e.g. in *Arthrobotrys oligospora* and *Monacrosporium haptotylum* [15,27,28]. This database contains microbial pathogenicity, virulence and effector genes which were experimentally characterized [29]. One good example is the *gEgh16/gEgh16H* gene family in *M*. *haptotylum*, for which a role in pathogenesis was described in *Blumeria graminis* and *Magnaporthe grisea* [30–32].

In this paper, we analyzed a *D*. *flagrans* SSP with a cysteine-rich region and named it CyrA (c*y*steine-rich protein A). The gene is highly upregulated in traps and the protein is secreted into *C. elegans* at a bulbous structure of the penetration peg. CyrA is important for fungal virulence and causes a reduction of the lifespan when expressed in *C*. *elegans*. This is the first example for the characterization of a SSP from a nematode-trapping fungus and paves the way for further studies to unravel the molecular interplay between fungi and nematodes.

## Results

### CyrA is a small-secreted protein with a cysteine-rich region

**Fig 1:**
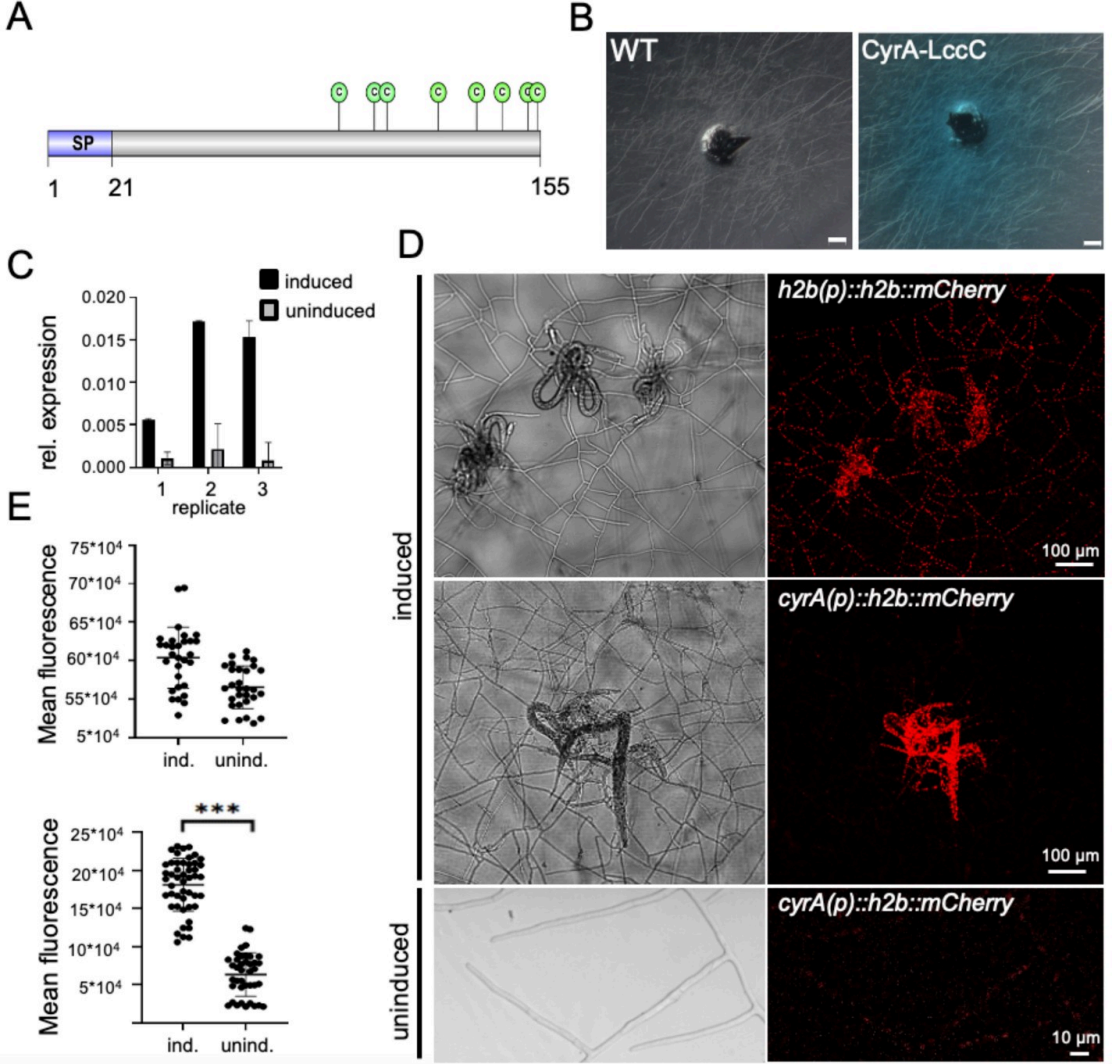
CyrA is a secreted cysteine-rich protein induced during trap formation. **(A)** Scheme of CyrA. The 155 amino acid long protein contains a 21 amino acid long signal peptide and a cysteine-rich region with eight cysteine residues. **(B)** The CyrA-LccC expressing *D*. *flagrans* strain sNH08 and the WT were grown on LNA with 1 mM ABTS for 48 hours. **(C)** Quantitative real time PCR analysis of *cyrA* expression in *D*. *flagrans* hyphae grown on LNA and in hyphae and traps co-cultivated for 24 h with *C*. *elegans*. The three biological replicates are displayed individually, and the error bar indicates the standard deviation of three technical replicates, P-value = 0.0342. The expression was normalized to actin. **(D)** Spatial analysis of *cyrA* expression using a transcriptional reporter assay. The *h2b-mCherry* reporter was expressed under the constitutive *h2b* (sNH14) or the *cyrA* promoter (sNH21), respectively. Pictures of traps and captured nematodes were taken after 24 h of co-incubation. Scale bar = 100 μm. Pictures of uninduced mycelium were taken after 24 h at 28°C. Scale bar = 10 μm. **(E)** The mean fluorescence of single nuclei of the reporter strains in traps (ind.) and vegetative mycelium (unind.) was measured in different pictures taken with the same settings using ImageJ (arbitrary units). *cyrA(p)* uninduced n = 39, *cyrA(p)* induced n = 48, *h2b(p)* uninduced n = 30, *h2b(p)* induced n = 30. P-value *h2b(p)* induced vs. *cyrA(p)* induced < 0.0001.

The analysis of the *D*. *flagrans* secretome revealed a large arsenal of SSPs that are potentially involved in the virulence against nematodes [15]. Since there are currently no detailed reports about the function of such proteins in the interaction between fungi and nematodes, we analyzed if candidate proteins were specific to nematophagous fungi, shown to be transcriptionally induced in RNAseq analyses of other nematophagous fungi and predicted to be involved in the attack by EffectorP [33]. One protein meeting these criteria is characterized in this analysis. The CyrA protein is composed of 155 amino acids and harbors a putative secretion signal at the N-terminus (1–21) **(Fig 1A)**. It contains no conserved domains, and orthologous proteins can be found only in the two nematode- trapping fungi *A*. *oligospora* and *M*. *haptotylum*. To test the functionality of the signal peptide, we fused the protein to *Aspergillus nidulans* Laccase C lacking its own signal peptide and expressed it in *D*. *flagrans*. If secreted, the laccase catalyzes the oxidation of the substrate ABTS in the medium to the more stable state of the cation radical [34]. This is indicated by a blue-green color. After 48 h incubation of the mutants on low nutrient agar (LNA) containing 1 mM ABTS at 28°C the colonies were surrounded by a blue-green color indicating the activity and secretion of the laccase into the medium and therefore functionality of the predicted signal peptide **(Fig 1B)**.

### *cyrA* is up-regulated during the interaction with *C*. *elegans*

If *cyrA* plays a role during the infection process, the expression of the gene could be specifically induced. To determine any differential expression of *cyrA* we performed quantitative real-time PCR (qRT PCR) of RNA extracted from uninduced mycelium and mycelium after co-incubation with *C*. *elegans* **(Fig 1C)**. Induction of the expression of the *cyrA* gene was observed after 24 h. The expression data varied between biological replicates with fold changes of 5.7 (± 0.7, n = 3), 7.9 (± 1.4) and 18.9 (± 2.6). These differences between the replicates reflect differences in trap numbers and/or trapped worms.

To obtain further evidence for the upregulation of *cyrA*, a reporter assay was used to visualize the activity of the *cyrA* promoter microscopically in hyphae **(Fig 1D)**. A fusion protein comprised of histone H2B and mCherry was expressed in *D*. *flagrans* under the control of the *cyrA* promoter. The H2B protein was used to target mCherry into nuclei to make it easier to distinguish any fluorescent signal from background fluorescence. Traps were induced by co-incubating the mycelium with *C*. *elegans* on LNA-microscopy slides, and a control was incubated without worms. Strong fluorescence of the nuclei was observed in traps as well as in trophic hyphae growing inside of captured *C*. *elegans*. Fluorescence decreased in mycelium further away from traps and was very weak in areas with no traps and in non-induced mycelium. The mean fluorescence of single nuclei was quantified using ImageJ **(Fig 1E)**. The same construct but under the control of the *h2b* promoter was used as a control for a constitutively expressed gene. In the corresponding mycelium all nuclei showed similar fluorescent signal intensities. This shows that the expression of *cyrA* is highly upregulated during trap formation and colonization of the worms.

### CyrA localizes in trap specific vesicles

**Fig 2:**
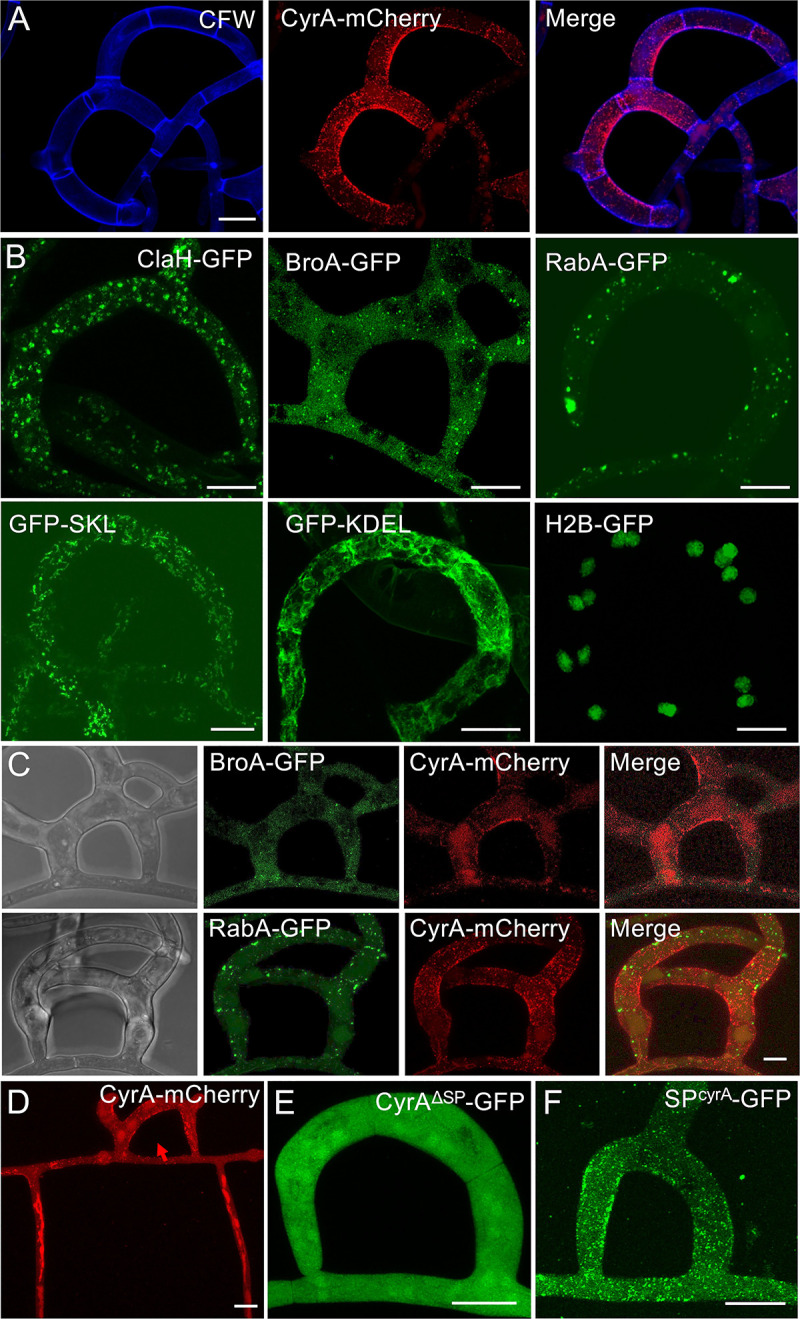
Localization of CyrA and different vesicles and organelles in traps of *D*. *flagrans*. **(A)** The CyrA-mCherry expressing strain sNH25 was co-incubated with *C*. *elegans* on LNA slides for 24 h at 28°C to induce traps. The cell wall was stained with calcofluor white (CFW). **(B)** Visualization of vesicles and organelles through GFP fusion proteins. Clathrin coated vesicles (ClaH-GFP, strain sNH66), exosomes (BroA-GFP, sNH52), endosomes (RabA-GFP, sNH29), peroxisomes (GFP-SKL, sNH16), the ER (GFP-KDEL, sNH22) and nuclei (H2B-mCherry, sNH14). Scale bars = 10 μm. **(C)** BroA-GFP and RabA-GFP, respectively, were expressed in the CyrA-mCherry expressing strain. Scale bars = 10 μm. **(D)** *cyrA-mCherry* was expressed under the constitutive *oliC*-promoter (sNH25) but the localization of the fusion protein in speckles was only apparent in traps (arrow). In the surrounding vegetative mycelium, CyrA-mCherry localized in vacuolar structures. **(E)** CyrA was expressed without its signal peptide sequence fused to GFP (strain sNH41). **(F)** The 21 amino acid long signal peptide of CyrA was fused to GFP (strain sNH42) and the construct was expressed under the constitutive *oliC* promoter.

To investigate the localization of CyrA during the infection, the protein was fused to GFP. The fusion protein was fully functional, because it rescued the virulence phenotype of a *cyrA-*deletion strain (see below). Over-expression of the CyrA-mCherry fusion protein with the constitutive *A*. *nidulans oliC* as well as under the native promoter revealed the localization of the protein in moderately dynamic speckles in the traps, mainly at the inner rim of the traps **(Fig 2A)**. The fusion protein was mainly visible when nematodes were already captured, just some empty traps showed a strong CyrA-mCherry signal. To investigate if the localization at the inner rim of the trap is a general occurrence in traps and whether these foci correspond to known vesicle populations or organelles, endosomes (RabA), exosomes (BroA), clathrin-coated vesicles (ClaH), peroxisomes (GFP-SKL), endoplasmic reticulum (GFP-KDEL) and nuclei (H2B-GFP) were visualized in the traps **(Fig 2B)**. All vesicle populations and organelles were evenly distributed as expected and the ER showed no unconventional arrangement. Peroxisomes were very abundant in the trap cells. None of these organelles showed the same localization pattern at the inner side of the trap observed in the CyrA-mCherry strain, as shown for exosomes and endosomes **(Fig 2C)**. These results indicate that the CyrA foci are trap-specific structures and suggest a trap-specific secretion mechanism that differs from conventional secretion at the tip. In non-induced mycelium the C-terminal fusion protein was either not visible or localized in vacuoles, suggesting degradation everywhere but in the traps **(Fig 2D)**.

Next, we constructed a strain expressing CyrA without its signal peptide fused to GFP **(Fig 2E)**. The fusion protein without SP localized in the cytoplasm of vegetative mycelium and traps, indicating a loss of trap identity. Because the SP appeared to carry the information for the correct protein localization, we fused only the SP (aa 1–21) to GFP. In this case, the SP was sufficient to reconstitute the initial localization at the inner side of the trap. In vegetative mycelium, diffuse localization in the cytoplasm was observed, but no vesicles were present without traps **(Fig 2F)**. This observation was interesting because one would expect GFP to localize in the ER after the signal peptide is cleaved off after entering the organelle. This indicated that the SP is sufficient to guide proteins to specific vesicles in the traps and that the secretory pathway in traps is distinct from the conventional one in vegetative hyphae.

### CyrA is secreted at the infection bulb

**Fig 3:**
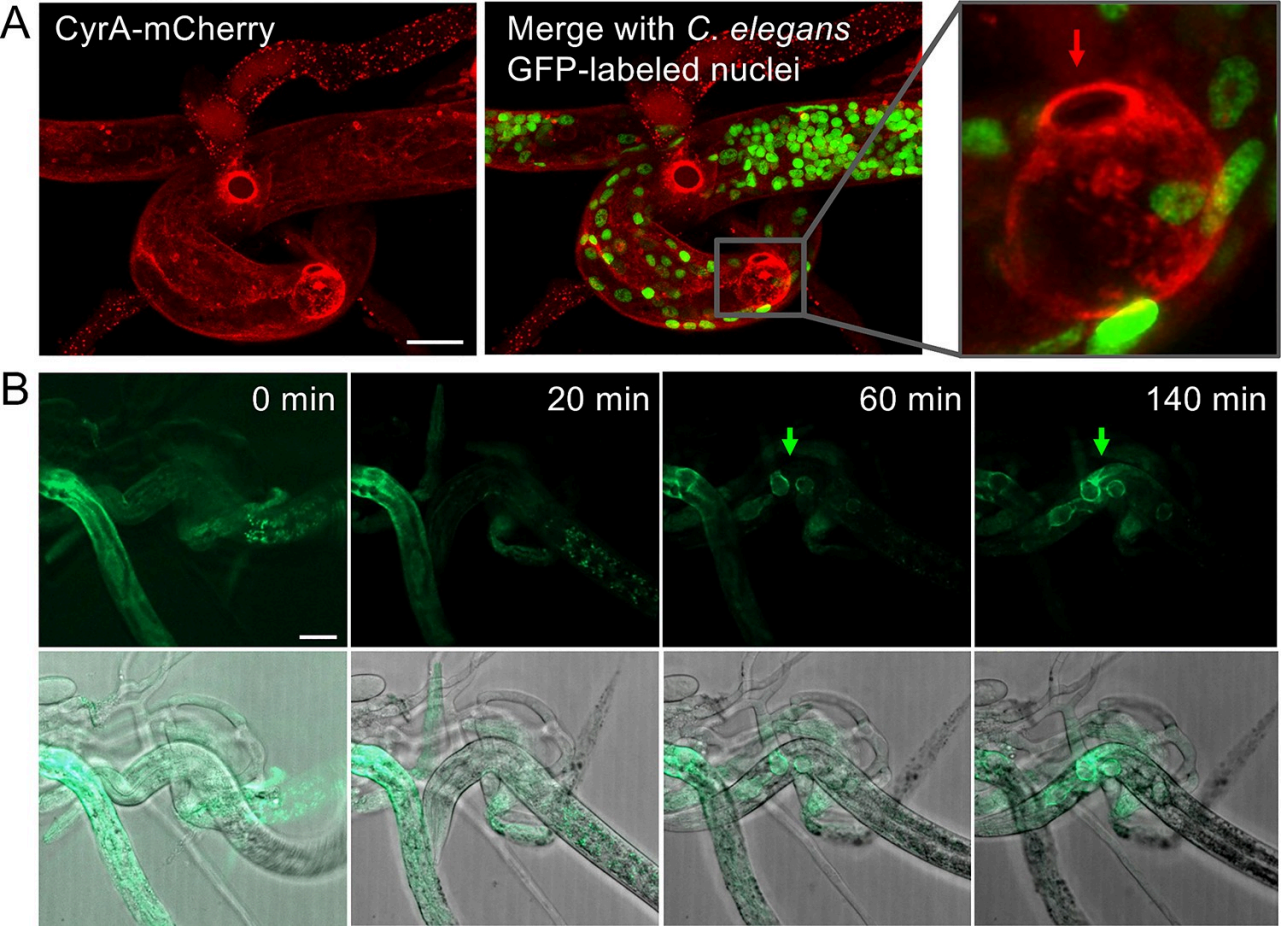
CyrA accumulates at the infection bulb during the attack. **(A)** The *cyrA(p)*::*cyrA*::*mCherry* expressing strain sNH65 was co-incubated for 24 h at 28°C with *C*. *elegans* Ban126 (*his-72p::his-72::GFP*). The enlargement shows the CyrA-mCherry localization in the infection bulb. The arrow points to the entry point of the fungus, above the infection bulb. **(B)** Time course of infection bulb formation (arrow) and accumulation of CyrA-GFP (strain sNH30). Pictures of *C*. *elegans* captured by *D*. *flagrans* were taken every minute and assembled into a movie sequence **(S1 Movie)**. Pictures of the indicated time points are displayed. Scale bars = 10 μm.

When nematodes were captured, the fusion protein (expressed from the *oliC* or the native promoter) accumulated in the infection bulb indicating secretion through this structure at the inside of the host **(Fig 3A)**. We used *C*. *elegans* Ban126, where nuclei are stained with GFP to distinguish between fungus and worm. Time-resolved microscopy revealed that the fusion protein emerged immediately after the nematode’s movement ceased and with the beginning of the penetration **(Fig 3B and S1 Movie)**. The signal of CyrA-mCherry remained in the bulbous structure even after the trophic hyphae grew from the site and spread through the nematode body. After 140 min, the nematodes were fully colonized and the accumulation at the bulb was still intact **(Fig 3B)**. When the construct was expressed under the control of the native promoter, the fusion protein was mainly visible at the infection bulb with fewer CyrA-GFP foci present in traps with captured nematodes. Only a few foci were present in empty traps indicating an important role of the protein during the initial steps of the entry of the fungus into *C*. *elegans*.

**Fig 4:**
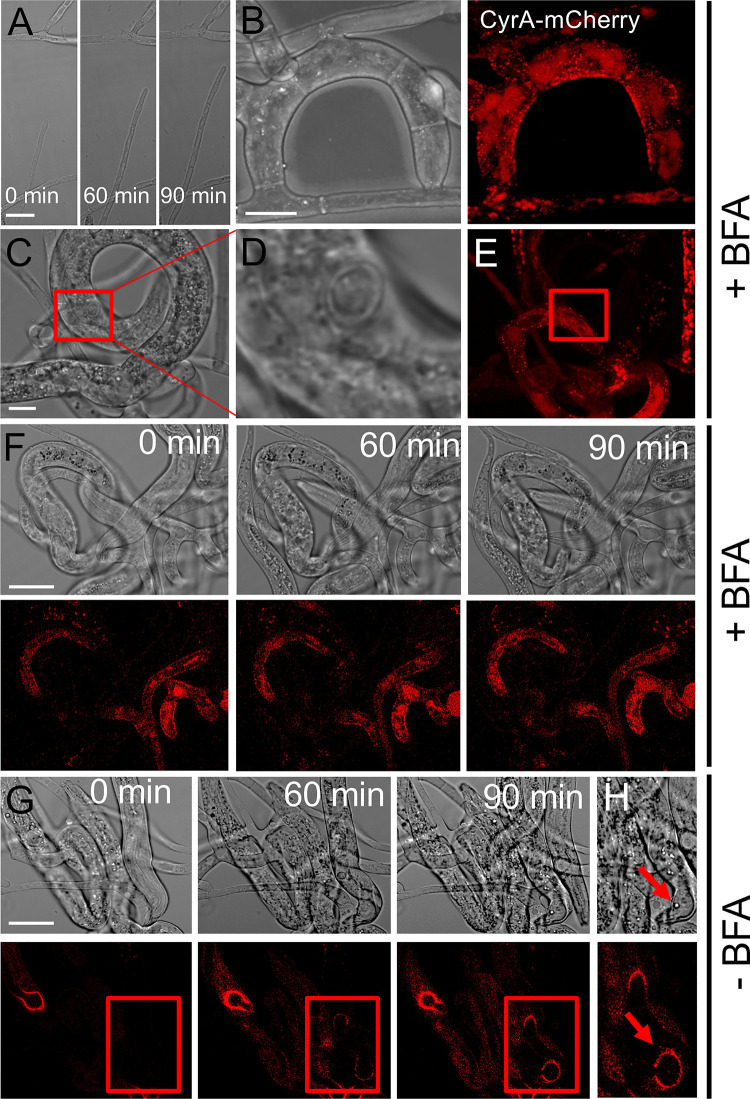
Brefeldin A treatment inhibits penetration and CyrA accumulation at the infection bulb. **(A)** *D*. *flagrans* expressing *cyrA(p)*::*cyrA*::*mCherry* (sNH65) was co-incubated with *C*. *elegans* on thin LNA slides for 24 h at 28°C. A 1 cm square was cut from the agar and placed upside down onto a 5 μl drop of 50 μg/ml BFA. A hypha after 60 and 90 min. **(B)** CyrA-mCherry localization in the trap after BFA treatment. **(C)** Observation of an infection site in the DIC channel (enlargement in **(D)**). The red box indicates the penetration site. **(E)** Localization of CyrA-mCherry after BFA treatment and penetration. The red box indicates the penetration site. **(F, G)** Time course of infection bulb formation and CyrA-mCherry distribution in the presence **(F)** and the absence **(G)** of BFA. **(H)** Enlargement of the formed infection bulbs after 90 min. The arrow points to the entry point of the fungus. Scale bars = 10 μm.

To check if CyrA secretion at the infection bulb is mediated by the conventional secretion pathway, the CyrA-mCherry expressing strain was treated with Brefeldin A (BFA) during the interaction **(Fig 4)**. BFA inhibits the transport from ER to Golgi by inhibition of ADP-ribosylation factor (Arf) activators [35]. Vegetative growth of the hypha was not impaired by the treatment **(Fig 4A)**. In traps that were already formed prior to BFA treatment, the localization of CyrA was not changed, the protein accumulated in spots at the inner side of the traps **(Fig 4B)**. Microscopic long-term observations of nematodes that were captured revealed that BFA treatment inhibited penetration of the worms **(Fig 4C–4F and S2 Movie)**. Most trapped nematodes did not show any penetration site even after 5 hours. In some cases, penetration sites were visible in the DIC channel but in these cases no CyrA-mCherry accumulation at the infection bulb was observed **(Fig 4F)**. The inhibition of the penetration is most likely due to the fact that many conventionally secreted proteins and enzymes are important for the penetration process. The lack of CyrA-mCherry accumulation at the infection bulb in nematodes that were likely already penetrated before BFA treatment could indicate that in these structures CyrA is secreted through the conventional secretion pathway dependent on the Golgi complex. Captured nematodes in the control, without BFA treatment, were penetrated after approx. 60 minutes and CyrA-mCherry localized at the infection bulb at this time **(Fig 4G)**.

### The exocyst complex is essential for CyrA localization in the infection bulb

**Fig 5:**
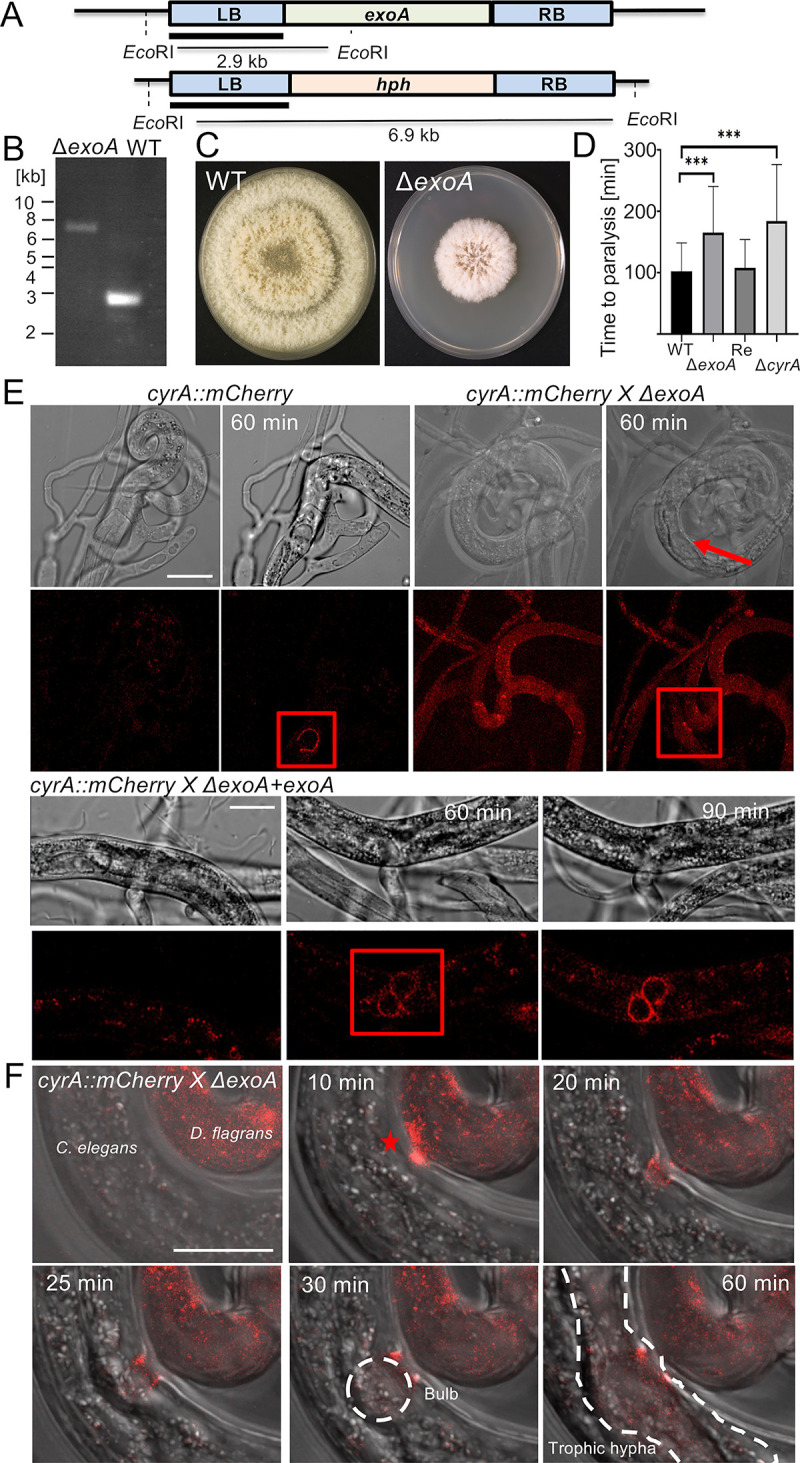
The exocyst component *exoA* is necessary for CyrA accumulation at the infection bulb. **(A)** Scheme of the deletion strategy for *exoA*. The gene was deleted via homologous recombination by flanking the hygromycin resistance cassette (hph) with 1 kb upstream (LB) and downstream (RB) flanks of *exoA*. The LB was used as a probe. **(B)** Southern-blot of genomic DNA of the *ΔexoA*-deletion mutant (strain sNH60) and WT using the probe indicated in **(A)**. **(C)** *D*. *flagrans* wildtype and the *ΔexoA-*deletion strain were grown for 7 days at 28°C on PDA. **(D)** Virulence assay with the *D*. *flagrans* wildtype (WT), the *ΔexoA*-deletion mutant, the complementation strain (Re) and the Δ*cyrA*-deletion mutant. The strains were co-incubated with a mixed *C*. *elgans* N2 population on thin LNA-slides for 24 h at 28°C. After trap formation the N2 worms were washed off the slides and a synchronized population of Ban126 L4-larvae was added to the traps just before the microscopical observation. Pictures of single traps were taken every 5 minutes for 20 hours. The time from the capturing event to full paralysis of the nematodes was taken. The statistical significance was calculated using a student’s t-test (*** = p-value< 0.0001 n [WT] = 35, n [*ΔexoA*] = 34, n [Re] = 17, n [*ΔcyrA*] = 27). The error bar indicates the standard deviation. **(E)** Visualization of CyrA-mCherry in wild type (sNH65), the *ΔexoA-*mutant strain (sNH60) and the complementation strain (*ΔexoA+exoA)*. The red box indicates the penetration area and the arrow points to the penetration site. **(S2 and S3 Movies) (F)** Enlargement of the interaction zone of the *cyrA-mCherry* expressing *ΔexoA-*mutant strain in **(E)**. Initially, CyrA accumulates at the infection site after 10 minutes (star). The infection bulb is established after 30 minutes but CyrA does not accumulate in the bulb, instead some signals are visible at the outer rim of the entrance site. After 60 min trophic hyphae have colonized the nematode but the fusion protein is not visible. Scale bars = 10 μm.

To further investigate the mechanism of virulence factor secretion in *D*. *flagrans* we deleted the *exoA* gene, coding for an orthologue of the exocyst complex component Exo70. We chose Exo70 for our analysis because it is already known to be involved in the secretion of cytoplasmic effectors in *M*. *oryzae* [23]. The *exoA* gene was deleted in a strain expressing CyrA-mCherry **(Fig 5A)**. The mutant was confirmed via Southern-blot analysis and showed a growth phenotype compared to the wild type **(Fig 5B and 5C)**. For the complementation, the mutant strain was transformed with a plasmid encoding the full-length gene under control of the native promoter. A virulence assay based on the long-term observation of single captured nematodes was performed, determining the time point of paralysis after the capturing event **(Fig 5D)**. Spores of the respective strains were inoculated on LNA microscopy slides together with the *C*. *elegans* N2 strain and incubated for 24 hours at 28°C to induce traps. The worms were then washed off and *C*. *elegans* BAN126 L4 larvae were added to the mycelium with already formed traps. Trapping networks were observed using a spinning disk confocal microscope and pictures were taken every 2 minutes for 12 hours. The time a worm got stuck in a trap was determined as starting point. The time when all movement ceased, was defined as the paralysis point. While the *D*. *flagrans* wildtype took 103 min [SD ± 46] to paralyze the L4-larvae and the complementation strain 108 min [SD ± 46], the *ΔexoA*-deletion strain paralyzed the worms slower with 166 min [SD ± 75]. This result demonstrates that the exocyst complex plays an important role in *D*. *flagrans* virulence. Microscopic analysis of CyrA-mCherry in the *ΔexoA*-deletion strain showed, that CyrA still localized in dynamic spots at the inner side of the traps but did not accumulate at the infection bulb immediately after penetration **(Fig 5E)**. This is also in accordance with the prolonged time to paralysis of the *cyrA*-deletion strain, which took 184 min [SD ± 92] and thus shows a similar impairment of virulence as the exoA mutant. Some CyrA-mCherry accumulations at the infection bulb were visible in old, already fully colonized nematodes over 5 hours after the penetration. While in the wildtype control and the complemented strain the fusion protein showed its characteristic localization after 60 min **(S3 Movie)**, this was not the case in the mutant strain even though penetration was clearly visible in the DIC channel. Magnification of the interaction zone showed that CyrA-mCherry accumulated at the infection site after 10 minutes but was not able to enter the nematode after the infection bulb was established, instead the protein seemed to remain at the outer edges of the infection site **(Fig 5F and S4 Movie)**. This observation indicates that the exocyst complex is necessary for the secretion of virulence factors at the infection bulb. It could play an important role in tethering the proteins to the specific membrane.

### Deletion of *cyrA* affects virulence

**Fig 6:**
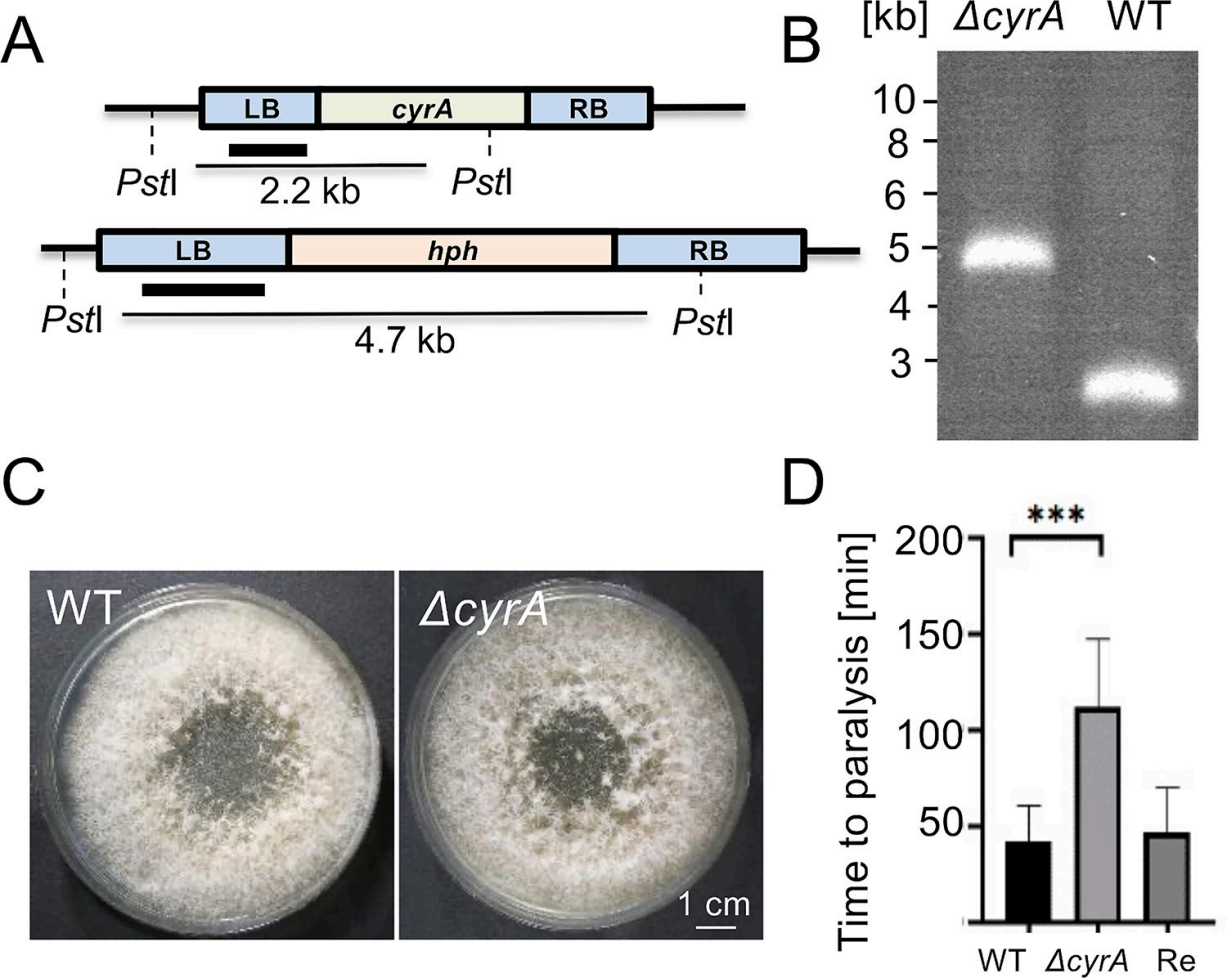
The *cyrA* gene is required for full virulence. **(A)** Scheme of the deletion strategy. The wild type *cyrA*-gene locus (upper panel) is replaced by the 1.8 kb hygromycin resistance cassette resulting in the *ΔcyrA-*mutant locus (lower panel). Southern-blot analysis using a 1 kb fragment from the LB as probe and the restriction enzyme *Pst*I for the digestion of the genomic DNA. **(B)** Confirmation of the *ΔcyrA* deletion using Southern-blot analysis. Genomic DNA was digested with *Pst*I, and the LB was used as probe. **(C)** The *D*. *flagrans* WT and the *ΔcyrA-*deletion strain (sNH11) were grown for seven days at 28°C on PDA. **(D)** Virulence assay with wild type (WT), the *ΔcyrA-*mutant strain and the strain re-complemented with the *cyrA* ORF (sNH27) under its native promoter (Rec.). Multiple traps were observed and the time from the capturing event to full paralysis was taken. Error bars indicate the standard deviation. A student’s t-test was performed for statistical analysis (*** = p-value < 0.0001; n [WT] = 29 n [KO] = 26 n [Rec] = 52).

To gain further insights into the molecular function of CyrA, *cyrA* was deleted via homologous recombination **(Fig 6A and 6B)**. The *cyrA*-deletion mutants displayed no vegetative growth phenotype, and trap formation was not affected **(Fig 6C)**. A virulence assay was performed with wild type *D*. *flagrans* and the *cyrA-*deletion strain using the *C*. *elegans* strain Ban126 as prey to visualize progression of the infection and cell death **(Fig 6D)**. Captured worms that are still alive displayed clear fluorescence in the nuclei. The GFP signal disappears when the cells die. We observed that indeed the nematode cells surrounding the trophic fungal hyphae died. There was no difference in the overall digestion process in the *ΔcyrA*-deletion strain as compared to wild type.

After the initial experiments, long-term observations of single captured nematodes were performed as described above. This experiment was carried out with wild type, the deletion mutant, and a re-complemented strain using *C*. *elegans* BAN126 L3-larvae. For the complementation, the mutant strain was transformed with a plasmid containing the full-length ORF including the regulatory regions, and death was determined by loss of the nuclear GFP signal. We also used a CyrA-GFP encoding plasmid for re-complementation to prove that the fusion protein is biologically active. *C*. *elegans* L3-larvae captured by *D*. *flagrans* wild type were paralyzed after 49 min [SD ± 27] and worms captured by the complementation strain after 62 min [SD ± 39]. The mutant strain paralyzed the worms within 95 min [SD ± 44]. The overall time until death of the mutant strain was with 206 min [SD ± 141] not significantly different compared to wild type, taking 146 min [SD ± 80] **(Fig 6D**). This indicates that CyrA directly or indirectly plays a role in either the penetration or the paralysis of the worms. The same assay was performed with L4-larvae, and the wildtype strain took 103 min [SD ± 46] to digest the larger nematodes whereas the *cyrA* mutant took 184 min [SD ± 92] **(Fig 5D).** This is in accordance with the prolonged time to paralysis in the *ΔexoA*-deletion strain, in which CyrA cannot accumulate at the infection bulb and therefore shows the same phenotype as the *ΔcyrA*-deletion strain.

### Expression in *C*. *elegans*

**Fig 7:**
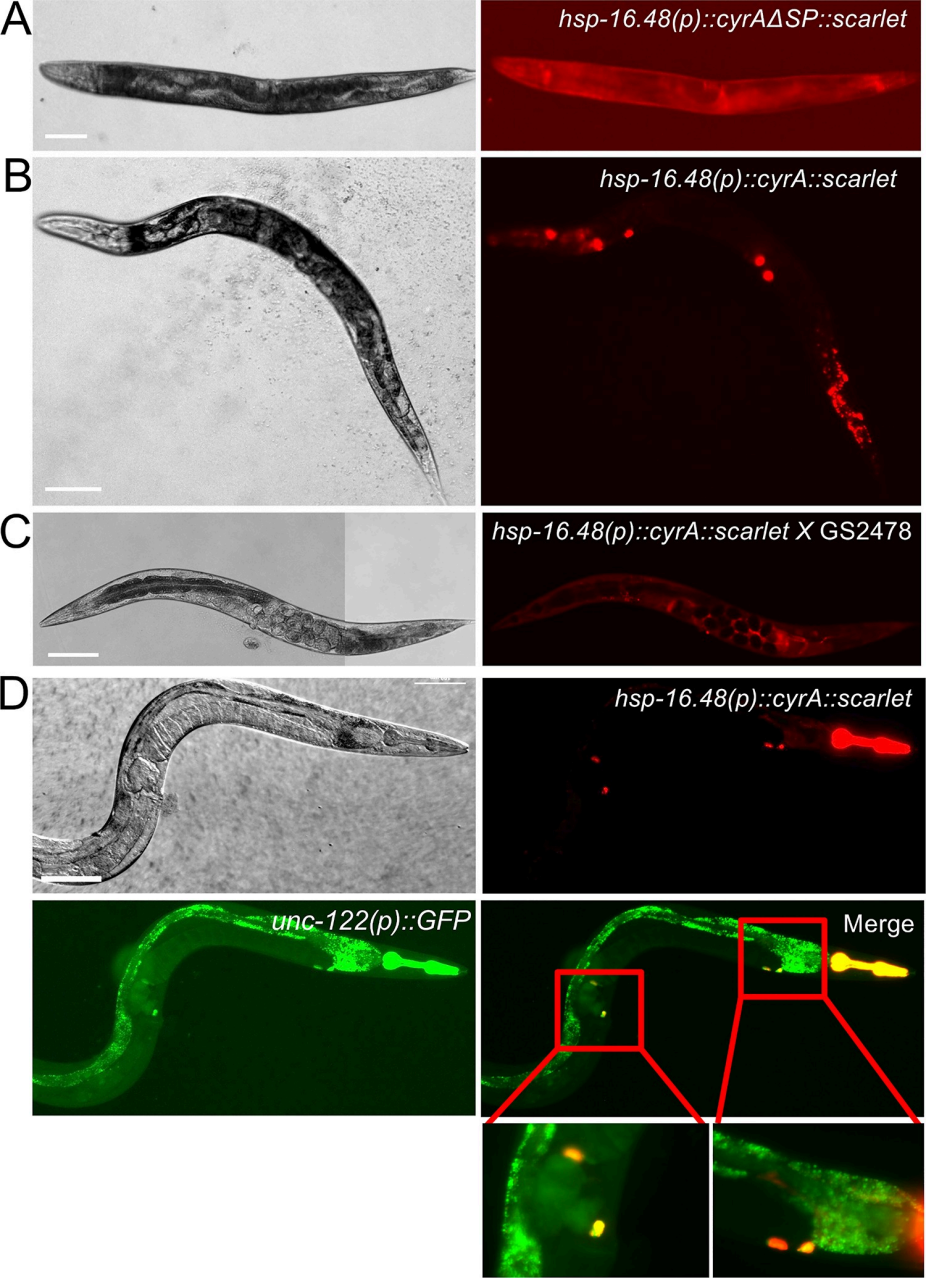
CyrA accumulates in coelomocytes in *C*. *elegans*. The *cyrA* gene fused to scarlet was expressed with (**A, C,** strain KIT02) and without (**B**, strain KIT04) its signal peptide sequence under the heat inducible promoter *hsp-16*.*48*. Expression was induced by heat shock at 37°C for 1 hour. **(C)** CyrA-Scarlet expressed with SP in the coelomocyte uptake deficient *C*. *elegans* strain *GS2478* (strain KIT26). Scale bars = 100 μm. **(D)** CyrA-Scarlet was co-expressed with the coelomocyte marker *unc-122(p)*::*GFP*. Co-localization is shown in the enlargements.

To investigate putative functions of CyrA during the infection and to identify possible targets of the virulence factor, we expressed *cyrA* in *C*. *elegans*. To be able to control *cyrA* expression, we expressed the gene without tag and in another approach fused it to the sequence of a fluorophore, respectively, under the heat inducible *hsp-16*.*48* promoter. This was carried out with and without the signal peptide sequence. Transgenic strains expressing *hsp-16*.*48(p)*::*cyrA*::*scarlet*::*unc-54UTR* and *hsp-16*.*48(p)*::*cyrA*::*unc-54UTR* as extrachromosomal array were generated with *myo-2p*::*GFP* as a co-marker leading to green fluorescence of the pharynx for selection **(Fig 7)**. A strain expressing only *hsp-16*.*48(p)*::*scarlet* was generated as a control. To induce gene expression, the strains were incubated at 37°C for one hour and induction of the promoter was observed under a fluorescent stereomicroscope for the fluorophore tagged versions. The scarlet signal was equally distributed, consistent with the *hsp-16*.*48* promoter expression profile, in the control strain and the strain lacking the signal peptide **(Fig 7A)**. Whereas the strain expressing *cyrA* with the signal peptide sequence showed accumulations of the fusion protein throughout the body, where two accumulations were observed close to the head, two mid-body and two in the tail region **(Fig 7B)**. This fluorescence pattern resembled the localization of coelomocytes, which are large, mesodermal scavenger cells in the pseudocoelomic cavity that endocytose fluid from the body cavity [36]. Therefore, the fusion protein secreted by the cells was likely being taken up by the coelomocytes. To confirm this, we expressed the same construct in a *C*. *elegans* strain impaired in coelomocyte function (**GS2478**; cup-8 mutant)[36]. The fluorescence of the fusion protein was now evenly distributed indicating that the observed localization pattern in the wild type did indeed show accumulation in the coelomocytes **(Fig 7C)**. To support this further, co-localization of *hsp-16*.*48(p)*::*cyrA*::*scarlet* with the coelomocyte marker *unc-122(p)*::*GFP* was shown **(Fig 7D**). Initially, it seemed possible that uptake of the fungal protein by the *C*. *elegans* cells interferes with its function when expressed with signal peptide, but the coelomocyte-deficient strain expressing *cyrA* without any fluorophore did not show any obvious phenotype. Although, in a system using the heat shock promoter the observation of any phenotype could be interfered by the dramatic impact of the heat shock itself, leading to behavioral and physical changes as well as differential gene expression of many factors also involved in immunity.

**Fig 8:**
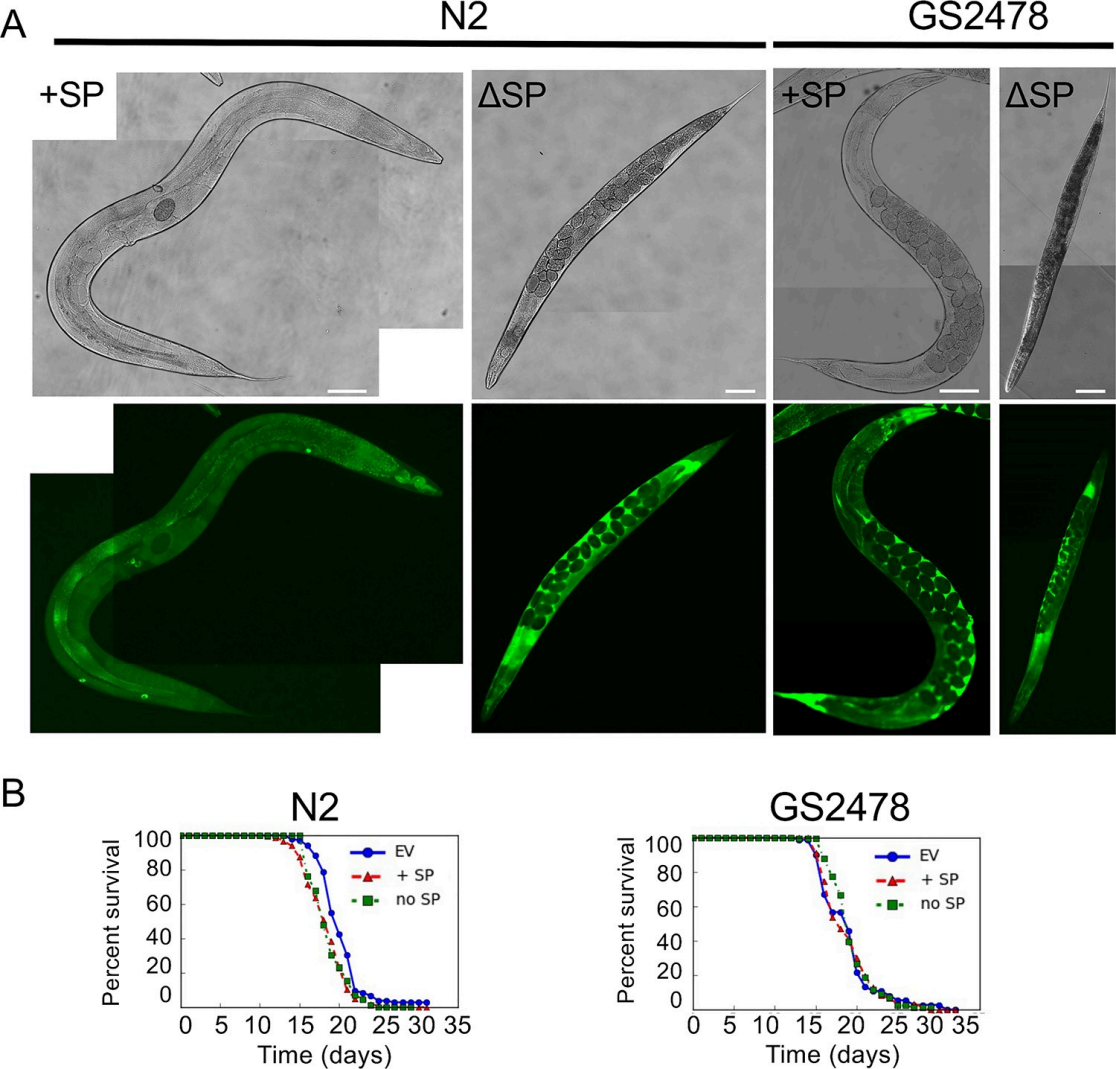
CyrA expression in the *C*. *elegans* hypodermis reduces the life span. **(A)** The *cyrA* gene fused to GFP was expressed with (strain KIT01) and without its signal peptide sequence (strain KIT23) in *C*. *elegans* N2 and GS2478 (*Δ*cup-8, strains KIT31/KIT29) under the hypodermal and adult specific promoter of the *col-19 gene*. Scale bars = 100 μm. **(B)** Survival curves of N2 and GS2478 expressing *cyrA* with (strain KIT22/KIT28) and without SP sequence (strain KIT18/KIT30). The empty vector (EV) was expressed in both strains as a control. Lifespan assays were performed on NGM plates with 150 mM FudR at 20°C. P—values log-rank-test N2: EV v.s. no SP = 0.0001; EV v.s. +SP = 1.2E-06; P—values GS2478: EV v.s. no SP = 0.9; EV v.s. +SP = 0.5.

Therefore, we expressed the same constructs under the control of the hypodermal and adult-specific *col-19* promoter in N2 and GS2478 **(Fig 8)**. During the infection, the fungus will penetrate the cuticle of the worm and encounters the hypodermis next. Therefore, the hypodermis could be a tissue possibly targeted by CyrA. Transgenic animals carrying extrachromosomal arrays of *cyrA-GFP* displayed green fluorescence in the hypodermis consistent with the expression profile of the *col-19* promoter. In the N2 background the secreted fusion protein accumulated in the coelomocytes **(Fig 8A)**. Life span assays of the strains without GFP fusion protein were conducted to observe any impact of the heterologous expression on the viability of the worms. Strains injected with the empty vector were co-injected with the same marker plasmids and used as control. The survival curve showed, that N2 worms expressing *cyrA* with and without the signal peptide sequence were short lived compared to the empty vector control **(Fig 8B)**. There was no effect on the lifespan of the GS2478 worms. This points towards a role of the scavenger cells in aging and immunity related signaling processes counteracting the effect of *cyrA* expression.

## Discussion

Fungal pathogens secrete virulence factors or effector proteins to overcome host defenses and to facilitate the infection [37–41]. We characterized the first small-secreted virulence factor in a predatory fungus, the nematode-trapping fungus *D*. *flagrans*. The analysis of this protein helped us to better understand the secretion process of this class of proteins, because it was mainly secreted at the infection or penetration bulb. The secretion mechanism and the role of the infection bulb will be discussed.

### Spatiotemporal localization of virulence factors

The localization of the fusion protein CyrA-mCherry revealed its accumulation at the infection bulb. This round structure is formed during the penetration, which requires high osmotic pressure and the secretion of lytic enzymes [42]. It is the first area of direct contact between fungal and nematode cells after the cuticle has been penetrated. The fact that the CyrA accumulation at the infection bulb was still visible in fully colonized nematodes suggests that this structure is not solely a side effect from the mechanical entry of the fungus but rather functionally specialized. Early observations of the infection process in *A*. *oligospora* already discussed the role of the infection bulb during the interaction [43]. Instead of the bulb solely representing the entry site of the fungus, *D*. *flagrans* might form an interaction zone similar to the *M*. *oryzae* biotrophic interfacial complex (BIC), which is a membrane rich structure formed inside the host [44,45]. The formation of the BIC is critical for the infection process of the fungus, and it is considered to be an active site of translocation of virulence factors into the host cell [46]. Thus, the *D*. *flagrans* infection bulb could act as a hub of the interaction zone from which virulence factors are secreted.

Most likely many of the proteins secreted at this site are crucial to overcome the first line of host defenses. Micrographs of later time points of the attack show no fusion protein surrounding the trophic hyphae that develop from the infection bulb. This enforces the hypothesis that CyrA is relevant during the early steps of the infection. Proteins that are important during the initial steps of the penetration before the formation of the bulb will most likely accumulate at the infection site rather than in the infection bulb. This observation shows that fluorescent protein fusions allow to specify the spatiotemporal occurrence of proteins secreted by *D*. *flagrans* and can therefore point towards their role during the attack.

Before a nematode has entered the trap, the fusion protein was observed in dynamic foci with accumulation at the inner side of the empty trap. Cytoplasmic vesicles and electron-dense particles were observed in early electron microscopical studies of *Dactylella ellipsospora* [47] and *A*. *oligospora* but there is a lack of new studies about the different vesicle populations found in the trapping structures of NTF. The accumulation of proteins at the inner side of the trap is intriguing. It seems like the cells know where to expect the prey even before any penetrating hypha is formed or a worm is captured. Interestingly, if a nematode adheres to the outside of a trap, the penetration peg is formed there and the accumulated protein shifts to the actual penetration site. Early reports describe the even distribution of electron-dense vesicles in *D*. *ellipsospora* knobs that move towards the point of contact with the nematodes only after adhesion [47]. Even though these knobs are different in shape, the underlying mechanisms could be the same but remain currently unknown.

When expressed under the constitutive *oliC* promoter the CyrA-mCherry fusion protein specifically localized in the traps and was degraded in the vegetative mycelium. The deletion of the signal peptide did not only abolish the presence of putative secretory vesicles in the traps, but also led to the cytoplasmic localization of the mature CyrA protein throughout the vegetative mycelium. Interestingly, the signal peptide was sufficient to guide GFP to the putative vesicles in the traps. No secretory vesicles were observed at the tips of vegetative hyphae instead GFP localized in the ER. This observation is intriguing because the signal peptide should be cleaved off after entering the ER, leading to GFP localization in the ER in traps as well as in vegetative hyphae. This reinforces the hypothesis of a specialized secretory pathway in trap cells. A similar phenomenon was observed for a SP-GFP fusion in mammalian endocrine cells where GFP localized in regulated secretory granules [48]. The comparison of the regulated secretion pathway of these mammalian cells with the one in the trap cells shows some parallels. Proteins secreted through this pathway are transported in a Golgi dependent manner [49]. Part of the vesicles is then tethered to the plasma membrane, and some are immobilized there while a reserve pool is retained at the actin cortex. After a stimulus, the releasable pool fuses to the plasma membrane and releases its content into the extracellular space [50]. The BFA treatment of the CyrA-mCherry expressing *D*. *flagrans* strain indicated dependance on the Golgi apparatus in our fungus as well. Further, the accumulation of CyrA at the inner side of the trap cells resembles the described localization of the secretory granules. While some of the vesicles in the traps are dynamic, others seem to be immobilized at the plasma membrane. Such immobilization would also explain that the accumulation of CyrA at the inner side of the trap was still visible after BFA treatment. These vesicles could be released upon penetration of a nematode, which represents the stimulus.

### The exocyst complex guides virulence factors to the correct membrane for secretion in the infection bulb

The octameric exocyst complex tethers secretory vesicles to the plasma membrane before fusion. After this exocyst guided docking, fusion is mediated by SNARE-proteins [51–54]. During the interaction between NTF and nematodes, many proteins need to be secreted in a spatiotemporally well-organized manner. While some proteins are secreted at the infection site, others are translocated at the infection bulb and again others are secreted by the trophic hyphae. Early studies already described the differential distribution of electron-dense microbodies and other small vesicles within traps, the infection bulb and trophic hyphae [55,56]. Here for example, many electron-dense microbodies were visible in the traps while just a few were found in the infection bulb. This specific secretion must be guided somehow and since the exocyst complex guides vesicles to the correct membrane it is highly likely that it plays a role in this process. The long-term observation of the CyrA-mCherry fusion protein in the *exoA*-deletion mutant during the penetration process showed that the protein was not able to accumulate in the infection bulb anymore while it still localized normal at the inner side of the traps. Interestingly, it shortly accumulated at the infection site just before penetration and stayed at the outer rims of the entrance site after establishment of the infection bulb. Thus, the protein was present in the trap and the penetrating hyphae but was not able to translocate into the infection bulb. Micrographs of the CyrA-mCherry fusion protein in wild type show that the protein usually localizes at the membrane in the infection bulb. Therefore, this shows that the exocyst complex is essential for tethering CyrA to the membrane of the infection bulb. A role of the exocyst complex in establishment of the epithelial polarity and localization of apical proteins was also shown in Drosophila [57]. Further, its role in pathogenesis and virulence factor secretion was shown in M. oryzae [25]. Here, Exo70 localizes at the appressorial ring indicating that it could play a role in tethering proteins to the correct membrane at this site. This localization resembles the localization of CyrA at the outer rim of the infection site in the *exoA* mutant suggesting that this would be where the protein is tethered to the correct membrane.

These results show that the exocyst complex plays a role in virulence factor secretion in NTF and they indicate that membrane identity is a crucial part of the spaciotemporal organization of proteins secreted during the interaction.

### Coevolution forges the sword of the *D*. *flagrans* attack

The *cyrA*-deletion strain was still able to trap and digest nematodes, but virulence was impaired as indicated by the prolonged time from the capturing event to paralysis. A rather small effect of the deletion of a single virulence factor is common in pathogenic interactions and is obvious when faced with the large and diverse weaponry of secreted proteins [58–60] that evolved in the arms race between host and pathogen. In 1973 Leigh Van Valen termed this concept the “Red Queen scenario” by making the analogy to Lewis Caroll’s novel *Through the Looking-Glass* in which the red queen says, **“**It takes all the running you can do, to keep in the same place”. Pathogens must develop new attack strategies to overcome host counter defenses and *vice versa*. The relatively minor impact of the *cyrA* deletion shows that this concept, which is very well described in the interaction of plant pathogens, also applies to the interaction between NTF and nematodes. Since the arms race between pathogens and multicellular hosts is not thoroughly explored yet, the interaction between NTF and nematodes, with more than 400 million years of co-evolution, represents a great model for such analyses [61,62]. Experimental coevolution experiments promise to help fight diseases, find new strategies for pest control, and find new drugs by understanding host defenses and virulence factors [63]. From the red queens’ perspective, it becomes clear that CyrA is just one of many needles, but a fungus needs a sword to kill a nematode. Thus, future analyses could investigate the existence of effector clusters to reveal the proteins essential for *D*. *flagrans* virulence. The deletion of a full effector gene cluster in *U*. *maydis* led to a severe virulence phenotype, while single deletions often only showed minor effects [64]. Further, novel genetic engineering approaches could be used to delete multiple virulence factor candidates at the same time to identify a set of core proteins responsible for *D*. *flagrans* virulence. Such an approach based on CRISPR/Cas was recently described for *B*. *cinerea* and could be adapted to NTF [65].

### Putative roles of the *C*. *elegans* coelomocytes during the *D*. *flagrans* attack

Like other invertebrates *C*. *elegans* solely relies on its basic innate immune system, but unlike others, the nematode does not possess any specialized immune cells. The only cells that might be remotely similar to Drosophila macrophage-like hemocytes for example are the coelomocytes which act as scavenger cells but do not seem to function through direct phagocytosis [66]. The observation of the uptake of secreted CyrA during the heterologous expression of the fungal gene raises the question of a possible role of the coelomocytes in fungal virulence factor uptake during the attack. A function of the coelomocytes during infection has already been indicated by their role in the regulation of the turnover of extracellular components through endocytosis of proteins in the pseudocoelomic cavity [36]. This process has been shown to be relevant during infection with pathogens because the optimization of extracellular proteostasis helps to sustain a systemic immune response and is implicated in intercellular signaling [67].

The fact that the reduction in lifespan was only apparent in the N2 strain background and not in the coelomocyte deficient strain could be due to their role in signal transduction. A role of the coelomocytes has already been described during the starvation response. During starvation, ASI neuron signaling leads to increased endocytosis by coelomocytes which in turn leads to NLP-7 signaling in neurons and induction of downstream genes in the intestine leading to lifespan extension. The coelomocytes are required for this lifespan extension during starvation [68]. That the effect of *cyrA* expression cannot be observed in the GS2478 strain could therefore point towards their role in multiple signal transduction pathways related to immunity and aging. Most likely, many of the functions of these scavenger cells are not yet described and should be analyzed in the future.

When CyrA was expressed in *C*. *elegans* with or without the signal peptide we observed a similar effect on the lifespan. This was surprising because the two proteins should localize at different places in the worm. However, this could be explained by cross tissue signaling [69]. It was also shown that neurons may regulate the innate immune response in distant tissues [70].

Taken together, the coelomocytes may play a role in the *C*. *elegans* defense during the *D*. *flagrans* attack. Further analyses of the responses of the immunity related processes in *C*. *elegans* during the interaction with NTF should help to shed light on these uncharacterized aspects of *C*. *elegans* immunity.

This is, to our knowledge, the first description of a role for a small-secreted protein in the interaction of the nematode-trapping fungus *D*. *flagrans* with *C*. *elegans*. The fact that deletion of the *cyrA* gene had only a small effect on virulence highlights the idea that the fungal attack probably requires the concerted action of many proteins perhaps along with low-molecular weight compounds to overcome the physical and other defense barriers of *C*. *elegans*. This work should be the groundwork for detailed analyses to unravel the interplay of virulence determinants in this fungal-animal interaction.

## Methods section

### Strains and culture conditions

#### Cultivation of the organisms

*D*. *flagrans* (CBS 349.94) was obtained from the CBS-KNAW culture collection (The Netherlands) and was cultured at 28°C on potato dextrose agar (PDA). *C*. *elegans* cultivation and synchronization was performed according to the worm book (doi/10.1895/wormbook.1.101.1). Strains are listed in **Tables 1 and 2**.

**Table 1:** *C*. *elegans* strains used in this study.

Strain	Genotype	Reference
N2	Wild type	University of Freiburg
Ban126	*zuIs178[his-72(p)*::*his-72*::*gfp]*	Dr. D. Bano, DZNE Bonn
GS2478	arIs37 I; *dpy-20*(e1282) IV; *cup-8*(ar466) V	Caenorhabditis elegans center, University of Minnesota
KIT01	*myo-2(p)*::*tdTomato col19(p)*::*SP*::*cyrA*::*GFP*	This work
KIT02	*myo-2(p)*::*GFP hsp16*.*48(p)*::*SP*::*cyrA*::*scarlet*	This work
KIT03	*myo-2(p)*::*GFP hsp16*.*48(p)*::*scarlet*	This work
KIT04	*myo-2(p)*::*GFP hsp16*.*48(p)*::*cyrA*Δ*SP*::*scarlet*	This work
KIT09	*myo-2(p)*::*tdTomato hsp(p)*::*cyrA+SP*	This work
KIT10	*myo-2(p)*::*tdTomato hsp(p)*::*cyrA*Δ*SP*	This work
KIT14	*myo-2(p)*::*GFP hsp16*.*48(p)*::*SP(cyrA)*::*Scarlet*	This work
KIT18	*myo-2(p)*::*tdTomato col-19(p)*::*cyrA*Δ*SP*	This work
KIT21	*myo-2(p)*::*GFP col-19(p)-Scarlet*	This work
KIT22	*myo-2(p)*::*tdTomato col-19(p)*::*cyrA+SP*	This work
KIT23	*myo-2(p)*::*tdTomato col-19(p)*::*cyrA*Δ*SP*::*GFP*	This work
KIT24	*myo-2(p)*::*GFP hsp(p)*::*SP*::*cyrA*::*scarlet x GS2478*	This work
KIT25	*myo-2(p)*::*GFP hsp(p)*::*cyrA*Δ*SP*::*scarlet x GS2478*	This work
KIT26	*myo-2(p)*::*tdTomato hsp(p)*::*cyrA+SP x GS2478*	This work
KIT27	*myo-2(p)*::*tdTomato hsp(p)*::*cyrA*Δ*SP x GS2478*	This work
KIT28	*myo-2(p)*::*tdTomato col-19P*::*cyrA+SP x GS2478*	This work
KIT29	*myo-2(p)*::*tdTomato col-19P*::*cyrA*Δ*SP*::*GFP x GS2478*	This work
KIT30	*myo-2(p)*::*tdTomato col-19P*::*cyrA*Δ*SP x GS2478*	This work
KIT31	*myo-2(p)*::*tdTomato col19(p)*::*SP*::*cyrA*::*GFP x GS2478*	This work
KIT32	*myo-2(p)*::*tdTomato hsp(p)*::*cyrA+SP*::*Scarlet unc-122(p)*::*GFP*	This work

**Table 2:** *D*. *flagrans* strains used in this study.

Strain	Genotype	Reference
sNH08	*cyrA-LccC^ΔAS1-18^* pNH10	This work
sNH11	*ΔcyrA* pNH12	This work
sNH14	*h2b(p)*::*h2b*::*mCherry*	This work
sNH16	*tubA(p)*::*GFP-SKL*::*gluC(t) pNH18*	This work
sNH21	*cyrA(p)*::*h2b*::*mCherry*::*tubT* pNH30	This work
sNH23	*oliC(p)*::*CyrA*::*GFP*::*gluC(t)* pNH21	This work
sNH25	*oliC(p)*::*CyrA*::*mCherry*::*gluC(t)* pNH24	This work
sNH27	*sNH11 x cyrA(p)*::*CyrA*::*CyrA(t)*::*trpC(p)*::*G418*::*trpC(t)*	This work
sNH29	*oliC(p)*::*GFP*::*rabA*::*gluC(t)* pNH38	This work
sNH30	*cyrA(p)*::*cyrA*::*GFP*::*gluC(t)* pNH32	This work
sNH41	*olic(p)*::*cyraΔSP*::*GFP*::*gluC(t)* pNH61	This work
sNH42	*olic(p)*::*SP*::*GFP*::*gluC(t)* pNH62	This work
sNH52	*olic(p)*::*broA*::*GFP; trpC(p)*::*G418*::*trpC(t)* pNH46	This work
sNH66	*claHP*::*claH*::*GFP*:: *trpC(t)* pNH97	This work
sNH22	*tubA(p)*::*dnaJ*::*GFP*::*KDEL*::*gluC(t)* pNH20	This work
sNH47	*cyrA*(p)::*cyrA*::GFP::gluC(t); tub(p)::G418 x sNH11	This work
sNH60	*ΔexoA*, *pNH73*	This work
sNH65	*cyrA(p)*::*cyrA*::*mCherry*::*gluC(t);tub(p)*::*G418*::*trpC(t)*	This work
sNH74	*exoALB*::*exoA*:*exAoRB*:: *tub(p)*::*Nat*::*trpC(t)*	This work

#### Trap induction

For trap induction around 1x10^4^ *D*. *flagrans* spores were inoculated on thin low-nutrient-agar (LNA) slides [15] (KCL 1g/L, MgSO_4_ - 7H_2_O 0.2g, MnSO_4_ - 4H_2_O 0.4mg, ZnSO_4_ - 7H_2_O 0.88mg, FeCl_3_ - 6H_2_O, 3mg, Agar 10g, pH 5.5). Around 100 individuals of *C*. *elegans* were added and co-incubation was carried out at 28°C in darkness for 12 hours.

#### Protoplast transformation of *D*. *flagrans*

Protoplast transformation was carried out as described [15]. 5x10^6^ protoplasts were transformed with 5–8 μg of DNA and transformants were incubated at 28°C for 4–7 days on PDA supplemented with 100 μg/ml hygromycin-B or 150 μg/ml geneticin (G418), respectively.

### RNA extraction and quantitative RT-PCR

For RNA extraction traps were induced by incubating 10^6^ *D*. *flagrans* spores on LNA covered with a cellophane membrane for 24 h at 28°C. 10^4^ individuals of a mixed *C*. *elegans* population were added to the membrane and co-incubated for 24 h to induce trap formation. For extraction of RNA from non-induced mycelium spores were treated the same without the addition of nematodes. The trap formation was microscopically observed, the mycelium was harvested and immediately frozen in liquid nitrogen. A micro pestle was used to grind the material.

For extraction of *C*. *elegans* RNA, worms were synchronized by treatment with an alkaline hypochlorite solution [71]. Alternatively, two adult *C*. *elegans* were transferred onto NGM plates and left for 12 hours to lay eggs. The adult worms were removed, and the offspring grown to the desired stage.

Total RNA was extracted with Trizol reagent (Invitrogen, Karlsruhe, Germany). DNase digestion was performed using the Turbo DNA-free Kit (Invitrogen, Karlsruhe, Germany) and the RNA was diluted to 50 ng/μl. The Luna One Step Kit (NEB, Germany) was used for the qRT-PCR analysis on an CFX Connect Real-Time PCR Detection System (Bio-Rad, Munich, Germany). Each reaction mixture contained 0.2 μM oligonucleotides **(Table 3)** and 100 ng of RNA in a 20-μl total volume. The gamma actin orthologue DFL_002353 was used as internal reference gene for normalization for *D*. *flagrans* and the *C*. *elegans* actin gene for nematode RNA. The qRT PCR was performed in 3 technical and 3 biological replicates.

**Table 3:** Oligonucleotides used in this study.

Name	Sequence (from 5’ to 3’)	Description
Backbone_Efi_for	TTAATTAACCGGGATCCAAGTG	Backbone amplification
Backbone_Efi_rev	GAATTCACTGGCCGTCGTTT	Backbone amplification efmov
h2b_NLS_for	ATGCCACCAAAAGCCGCC	Backbone amplification
ClaHp_tglucOL_for	ATGCTCTTTCCCTAAACTCCCCCCATCTCGGTTCCTCCCGCTT	ClaH-GFP fusion
ClaH_gfpOL_rev	GATTACTTACCTCACCCTTGGAAACGAATCCACGATAACCAGTAG	ClaH-GFP fusion
CyrA_genomic_for	AATTGGCAGCATCGATACTCG	Cornfirmation of the KO
CyrA_genomic_rev	CCATAACGCCAAGCGCTT	Cornfirmation of the KO
trpcT_rev	TGGGGGGAGTTTAGGGAAAG	C-terminal *cyrA* mCherry *cyrA*(p)
cyraP_trpctOL_for	ATGCTCTTTCCCTAAACTCCCCCCATGCATTCCAATCACTCAACCC	C-terminal *cyrA* mCherry *cyrA*(p)
cyraP_cyraOL_rev	GAACGATAGTGCTGAGGAGCTGCATTTTCGACAGTATTTGTGAAAAGAAGT	C-terminal *cyrA* mCherry *cyrA*(p)
*cyrA*_noSP_oliOL_for	CTCCATCACATCACAATCGATCCAAATGAACCCCAATGTATACGACTCAT	C-terminal CyrA wo SP
*cyrA*_nostop_gfpOL_rev	GATTACTTACCTCACCCTTGGAAACGTAGCACTTTTCGCACAAAGT	C-terminal CyrA wo SP
CyrA_rev	CTTAATTAAGTAGCACTTTTCGCA	C-terminal mCherry fusion of *cyrA*
tGluC_for	CGTATGTAGATAAGATGTATGATT	C-terminal mCherry fusion of *cyrA*
cherry_cyraOL_for	GTGCGAAAAGTGCTACTTAATTAAGATGGTAAGCAAGGGCGAGGT	C-terminal mCherry fusion of *cyrA*
cherry_glucTOL_rev	TAATCATACATCTTATCTACATACGCTAAGCGGCCGCTTTGTAGA	C-terminal mCherry fusion of *cyrA*
mcherry_h2bOL_rev	TTTCGGCGGCGGCTTTTGGTGGCATAGCGGCCGCTTTGTAGAG	C-terminal_promoter fusion
h2b_cherryOL_for	GGATGAACTCTACAAAGCGGCCGCTATGCCACCAAAAGCCGCC	C-terminal_promoter fusion
h2b_tubtOL_rev	CAAAGTAGGAATGACATCAGATATCTATTTGGCAGACGAGGAAGAGTA	C-terminal_promoter fusion
BroA_asc_for	TATGGCGCGCCATGGCCGGACTTCACCAAG	C-terminal GFP fusion BroA
BroA_pac_rev	GCGTTAATTAATCTCCAAGATTGAAGACCTGAC	C-terminal GFP fusion BroA
hsp16.48P_rev	TTCTTGAAGTTTAGAGAATGAACAG	*CyrA under hsp-16*.*48 in C*. *elegans*
Scar_for	GTC AGC AAG GGA GAG GCA	*CyrA under hsp-16*.*48 in C*. *elegans*
unc54_for	GAGCTCCGCATCGGCCGC	*CyrA under hsp-16*.*48 in C*. *elegans*
miniPPF_rev	GGGCCCTGTGAAATTGTTATCCG	*CyrA under hsp-16*.*48 in C*. *elegans*
hspP_ppfOL_for	AGCGGATAACAATTTCACAGGGCCCGCTGGACGGAAATAGTGGTAA	*CyrA under hsp-16*.*48 in C*. *elegans*
hspP_uncOL_rev	TAACTGCCTCTCCCTTGCTGACCATTTCTTGAAGTTTAGAGAATGAACAG	*CyrA under hsp-16*.*48 in C*. *elegans*
scr_hspPol_for	CTGTTCATTCTCTAAACTTCAAGAAATGGTCAGCAAGGGAGAGG	*CyrA under hsp-16*.*48 in C*. *elegans*
scr_uncOL_rev	GACAGCGGCCGATGCGGAGCTCTTACTTGTAGAGCTCGTCCATTC	*CyrA under hsp-16*.*48 in C*. *elegans*
hspP_cyraOL_rev	GAACGATAGTGCTGAGGAGCTGCATTTCTTGAAGTTTAGAGAATGAACAG	*CyrA under hsp-16*.*48 in C*. *elegans*
*cyrA*_hspPol_for	CTGTTCATTCTCTAAACTTCAAGAAATGCAGCTCCTCAGCACTAT	*CyrA under hsp-16*.*48 in C*. *elegans*
*cyrA*noSTOP_scrOL_rev	TGATAACTGCCTCTCCCTTGCTGACGTAGCACTTTTCGCACAAAGTC	*CyrA under hsp-16*.*48 in C*. *elegans*
scr_cyraOL_for	TAAGACTTTGTGCGAAAAGTGCTACGTCAGCAAGGGAGAGGCA	*CyrA under hsp-16*.*48 in C*. *elegans*
ppf37_MCS_rev_new	CTCGAGGAATTCCTGCAGG	*CyrA under hsp-16*.*48 in C*. *elegans*
*cyrA*+SP_col19pOL_for	TTTAGAAAGACATCAGTTCATCAACATGCAGCTCCTCAGCACTAT	*CyrA-GFP fusion in C*. *elegans*
emGFP_*cyrA*nostop_for	TAAGACTTTGTGCGAAAAGTGCTACTCAGGTGGATCTGGAGGC	*CyrA-GFP fusion in C*. *elegans*
emGFP_UTRol_rev	GATGACAGCGGCCGATGCGGAGCTCTCATTTGTAAAGTTCATCCATTCC	*CyrA-GFP fusion in C*. *elegans*
*cyrA*_col19pOL_for	TTTAGAAAGACATCAGTTCATCAACATGAACCCCAATGTATACGAC	*CyrA-GFP fusion in C*. *elegans*
CyrA_stop_uncOL_rev	GATGACAGCGGCCGATGCGGAGCTCTTAGTAGCACTTTTCGCACAAAGT	*CyrA-GFP fusion in C*. *elegans*
col19_mcsOL_for	AGATATCCTGCAGGAATTCCTCGAGACGTACCATTATTCGAGACAAC	*CyrA-GFP fusion in C*. *elegans*
col19_cyraSPOL_rev	GAACGATAGTGCTGAGGAGCTGCATGTTGATGAACTGATGTCTTTCTAA	*CyrA-GFP fusion in C*. *elegans*
ppf37_MCS_rev	CCTGCAGGAATTCCTCGAG	*CyrA-GFP fusion in C*. *elegans*
SP*cyrA*_rev_P	GGGGTTGGCCATGGCGAC	*cyrA*P::SP-GFP in pNH32/pNH21
dflGPD_bbOL_for	TCGAGTTTTTCAGCAAGATGGATCCGGTATCTATTACATTGCATTGC	*D*. *flagrans gpd promoter g418*
dflGPD_g418OL_rev	CGTGCAATCCATCTTGTTCAATCATTTTGAATTATTGACTTTTGTCGAG	*D*. *flagrans gpd promoter g418*
G418_for	ATG ATT GAA CAA GAT GGA TTG CA	*D*. *flagrans gpd promoter g418*
*cyrA*_pjOL_LB_for	GATGGCTCGAGTTTTTCAGCAAGATTTTCCTAAAGCCAAGTGTTCC	Deletion of *cyrA*; Δ*cyrA*
*cyrA*_hphOL_LB_rev	GACCTCCACTAGCATTACACTTATCGTAGCCATTGTTGTCATAG	Deletion of *cyrA*; Δ*cyrA*
*cyrA*ol_Hyg_for	CTATGACAACAATGGCTACGATAAGTGTAATGCTAGTGGAGGTC	Deletion of *cyrA*; Δ*cyrA*
*cyrA*ol_Hyg_rev	CAAAGTCTTAATACCACTGACACAGATGTTTGGGGGGAGTTTAGGGAAAG	Deletion of *cyrA*; Δ*cyrA*
*cyrA*_hphOL_RB_for	GCTCTTTCCCTAAACTCCCCCCAAACATCTGTGTCAGTGGTATTAAG	Deletion of *cyrA*; Δ*cyrA*
*cyrA*_pjOL_RB_rev	ATTGTAGGAGATCTTCTAGAAAGATTGGAATCCCGGCTCGTTTATTC	Deletion of *cyrA*; Δ*cyrA*
CyrA_woSP_asc_for	GGCGCGCCATGAACCCCAATGTATACGAC	GFP fusions CyrA
CyrA_pac_rev	TTAATTAATTAGTAGCACTTTTCGCACAAA	GFP fusions CyrA
CyrA_asc_for	GGCGCGCCATGCAGCTCCTCAGCACTA	GFP fusions CyrA
CyrA_nostop_pac_rev	TTAATTAAGTAGCACTTTTCGCACAAAGT	GFP fusions CyrA
*cyrA*_AscI_for	AATGGCGCGCCATGCAGCTCCTCAGCACTAT	Laccase-Assay; CyrA - Laccase; *Asc*I
*cyrA*_NheI_rev	ATAGCTAGCGTAGCACTTTTCGCACAAAGT	Laccase-Assay; CyrA - Laccase; *Nhe*I
mCherry_Tub(t)OL_rev	GCAAAGTAGGAATGACATCAGATATCTAAGCGGCCGCTTTGTAGAG	Promotorfusion full h2b
bcmCherry_for	ATGGTAAGCAAGGGCGAGG	Promotorfusion h2b-mCherry
h2b_cyrapOL_for	CTTCTTTTCACAAATACTGTCGAAAATGCCACCAAAAGCCGCC	Promotorfusion h2b-mCherry
h2b_mcherryOL_rev	GATTACTTACCTCGCCCTTGCTTACCATTTTGGCAGACGAGGAAGAGT	Promotorfusion h2b-mCherry
dfh2b_for	ATGCCACCAAAAGCCGCC	Promotorfusion h2b-mCherry
*cyrA*_qPCR_for	TCTTGCCGTCCCTCTTTTCA	qRT-PCR *cyrA*
*cyrA*_qPCR_rev	TCCGGTATCTTTGGCACCAT	qRT-PCR *cyrA*
rab_asc_for	ATGGCGCGCCATGGCTGAAGGCGGTCCA	RabA-GFP
rab_pac_rev	GCTTAATTAACTAACAAGCACAACCGTCCT	RabA-GFP
*cyrA*_LB_pjetOL_for	GATGGCTCGAGTTTTTCAGCAAGATTTTCCTAAAGCCAAGTGTTCC	Recomplementation *cyrA* KO
*cyrA*_RB_trpcTol_rev	GTTGACCTCCACTAGCATTACACTTTGGAATCCCGGCTCGTTTA	Recomplementation *cyrA* KO
pjetBB_rekomb_rev	ATCTTGCTGAAAAACTCGAGC	Recomplementation *cyrA* KO
trpcP_for	AAGTGTAATGCTAGTGGAGGT	Recomplementation *cyrA* KO
cyra_mid_rev	TGTTGTCATAGGCCTTGTTG	Sequencing of CyrA fusion constructs
*cyrA*_RB_sonde_for	AGTTACGTATCTCGTGAGCGA	Southern-analysis probe; RB *cyrA*
*cyrA*_RB_sonde_rev	ATCTTTTATGCTGTACGTCCAAC	Southern-analysis probe; RB *cyrA*
olicP_efiOL_for	TCACAATCGATCCAACCGGCGCGCCATGCGCGGTCTCCTCACTTA	SP-CyrA
cyraSP_GFPol_rev	GATTACTTACCTCACCCTTGGAAACGGGACCGGCGAGGGCC	SP-CyrA
olicP_efiOL_for	TTGTAAAACGACGGCCAGTGAATTCTGCAGCTGTGGAGCCGCATT	SP-GFP fusion
cyrASP_GFPol_rev	TACTTACCTCACCCTTGGAAACCATGGGGTTGGCCATGGCGAC	SP-GFP fusion
Exo_LBol_for	GATGGCTCGAGTTTTTCAGCAAGATTACCATTGGATGATGACGTTGTT	*exoA* deletion
Exo_Lbol_rev	GTTGACCTCCACTAGCATTACACTTGGTCGGTGCTTTTTATTGTCC	*exoA* deletion
hph_exoOL_for	GAGGGGACAATAAAAAGCACCGACCAAGTGTAATGCTAGTGGAGGT	*exoA* deletion
hph_exoOL_rev	AAGCAGAAGAGACAAAACCCCAAGATGGGGGGAGTTTAGGGAAA	*exoA* deletion
Exo_Rbol_for	ATGCTCTTTCCCTAAACTCCCCCCATCTTGGGGTTTTGTCTCTTCT	*exoA* deletion
Exo_RBol_rev	ATTGTAGGAGATCTTCTAGAAAGATGGCTACGCATGCTATGAAG	*exoA* deletion
Exo_LB_for	TAC CAT TGG ATG ATG ACG TTG	*exoA* deletion
Exo_RB_rev	GGCTACGCATGCTATGAAG	*exoA* deletion

### Plasmid construction

All plasmids are listed in **Table 4**. For the ABTS-assay, the modified vector pOF018 was used for the expression of the *A*. *nidulans lccC* gene (AspGD identification AN5397) under the constitutive *A*. *nidulans* glyceraldehyde-3-phosphate dehydrogenase (*gpdA)* promoter and as positive control for laccase activity as described [15]. The *cyrA* gene sequence was fused to the 3’ end of the laccase C by insertion into the vector backbone using the restriction enzymes *Nhe*I and *Asc*I. Standard protocols were used for *E*. *coli* transformation and plasmid isolation [72].

**Table 4:** Plasmids used in this study.

Name	Description	Reference
pCFJ90T	myo-2p::tdtomato	A. Dillin, UC Berkeley
pMyo-2-EGFP	myo-2p::GFP	R. Baumeister, Uni. Freiburg
pNH10	*gpd(p)::cyrA-Laccase^ΔAS1-18^ trpC(p)::hph::trpC(t) (hph)*	This work
pNH12	*ΔcyrA x pJET1*.*2; hph*	This work
pNH21	*oliC(p)*::*cyrA*::*GFP*::*gluC(t); hph*	This work
pNH22	*oliC(p)*::*GFP*::*cyrAΔSP*::*gluC(t); hph*	This work
pNH24	*oliC(p)*::*cyrA*::*mCherry*::*gluC(t; hph*	This work
pHN29	*cyrA(p)*::*cyrA*::*CyrA(t); trpC(p)*::*G418*::*trpC(t)*	This work
pHN30	*cyrA(p)*::*h2b*::*mCherry*::*tubT; hph*	This work
pNH32	*cyrA(p)*::*cyrA*::*GFP*::*gluC(t); hph*	This work
pNH38	*oliC(p)*::*GFP*::*rabA*::*gluC(t); hph*	This work
pNH20	*tubA(p)*::*dnaJ*::*GFP*::*KDEL*::*gluC(t); hph*	This work
pNH46	*olic(p)*::*broA*::*GFP; trpC(p)*::*G418*::*trpC(t)*	This work
pNH61	*olic(p)*::*cyraΔSP*::*GFP*::*gluC(t); hph*	This work
pNH62	*olic(p)*::*SP*::*GFP*::*gluC(t); hph*	This work
pNH72	*cyrA(P)*::*cyrA*::*GFP*::*gluC(T); tub(P)*::*G418*::*trpC(t)*	This work
pNH48	*col19(p)*::*SP*::*cyrA*::*GFP*::*unc-54UTR*	This work
pVW23	*h2b(p)*::*h2b*::*mCherry; hph*	[15]
pNH58	*hsp16*.*48(p)*::*cyrA+SP*::*scarlet*::*unc-54UTR*	This work
pNH59	*hsp16*.*48(p*::*scarlet*::*unc-54UTR*	This work
pNH97	*claHP*::*claH*::*GFP*::*gluC(t)*	This work
pNH106	*col-19P*::*cyrAnoSP+GFP*::*gluC(t); hph*	This work
pNH103	*col-19P-Scarlet*::*unc-54UTR*	This work
pNH104	*col-19P*::*CyrA*::*unc-54UTR*	This work
pNH13	*oliC(p)*::*GFP-SKL*::*gluC(t); hph*	This work
pNH38	*oliC(p)*::*bcGFP*::*rabA*::*gluC(t); hph*	This work
pNH90	*col19(p)*::*cyrA*::*unc-54UTR*	This work
pNH63	*hsp16*.*48(p)*::*cyrAΔSP*::*scarlet*	This work
pNH109	*exoALB*::*exoA*:*exAoRB*:: *tub(p)*::*Nat*::*trpC(t)*	This work

To create the promotor fusion of the *cyrA* promoter fused to the histone H2B sequence and a C-terminal mCherry fusion a 1.5 kb fragment upstream of the ORF of DFL_006380 was amplified by PCR, using *D*. *flagrans* genomic DNA as template. The vector pNH31 consisting of the H2B gene (DFL_000203) under its native promoter fused to mCherry was amplified as well and used as a backbone [15]. The two fragments were assembled via Gibson assembly.

For the C-terminal GFP fusion protein of CyrA the gene was amplified with a forward primer containing an *Asc*I restriction site and start codon and a reverse primer containing a *Pac*I restriction site. The amplicon was cut with the respective enzymes and ligated into a plasmid that contains the hygromycin-B resistance cassette *hph* [73] and the GFP gene under control of the constitutive *A*. *nidulans oliC* promotor and the glucanase terminator of *B*. *cinerea* (*oliC*(p)::*cyrA*::GFP::*gluC*(T)) with a pJET1.2 backbone resulting in the plasmid pNH21. For the C-terminal GFP fusion under the native *cyrA* promoter a 1.5 kb sequence upstream of the ORF was amplified as well as pNH21 as vector backbone and assembled via Gibson assembly resulting in pNH32. The GFP fusion without the signal peptide (pNH61) was performed in the same manner using pNH21 as a backbone, but the gene was amplified without the signal peptide sequence. For the CyrA-mCherry fusion the fluorophores were exchanged using Gibson assembly.

For the localization of the different organelles, pNH21 was used as a backbone and the respective gene sequences were inserted via Gibson assembly (BroA (dfl_002479), ClaH (dfl_009034), RabA (dfl_005994)).

The *cyrA* and the *exoA* gene were deleted by homologous recombination. 1.5 kb flanks homologous to the 5’ and 3’ region of the corresponding gene were amplified by PCR, using *D*. *flagrans* genomic DNA as template. The hygromycin-B resistance cassette *hph* was amplified using pFC332 [73] as template. The PCR-fragments were assembled into the pJET1.2 vector (Thermo Fisher) using Gibson Assembly. The fragments were amplified with Phusion polymerase (NEB) using the manufacturers recommended protocol and contained 25 bp overlapping regions to the neighbouring fragment. For the complementation strain, the *exoA* ORF 1-kb upstream and 0.5 downstream regulatory regions were cloned into a vector with the nourseothricin resistance cassette for selection.

To reintroduce the functional *D*. *flagrans cyrA* gene into the Δ*cyrA* deletion strain, the whole *cyrA* gene, including its 1.5-kb upstream and 0.5 downstream regulatory regions, was amplified via PCR, using *D*. *flagrans* genomic gDNA as template. To select for transformants the geneticin G418 resistance cassette was used. In a cloning step, the G418 resistance cassette was assembled between the *trpC*(p) promoter and *trpC*(t) terminator fragment of pVW23 [15]. Lastly, the G418 resistance cassette and the *cyrA* complement were assembled into the pJET1.2 vector using Gibson Assembly.

For the expression of *cyrA* in *C*. *elegans*, the open reading frame was cloned from cDNA, with or without the predicted signal peptide (AA 1–21), into the pLZ29 backbone [74] using Gibson assembly. For the localization of the constructs CyrA was C-terminally tagged with GFP or Scarlet. The constructs were expressed under the hypodermal *col-19* promoter, or the heat shock promoter *hsp-16*.*48*.

### Generation of transgenic *C*. *elegans* strains

Plasmids harboring the *cyrA* fusion constructs were injected at a concentration of 5 ng/μl into wildtype worms (N2) with a pharyngeal co-injection marker (myo-2p::tdTomato or myo2p::GFP) 5 ng/μl and 1 kb ladder (Eurofins) as filler DNA. Co-injection marker positive transformants were selected.

### ABTS-assay

For the ABTS-assay 1x10^4^ spores of *D*. *flagrans* transformants were grown on LNA containing 1 mM ABTS (2,2‘-azino-bis-[3-ethylthiazoline-6-sulfonate]) at 28°C. Laccase activity was correlated with the formation of blue-green *ABTS*• visible after 24–48 h.

### Brefeldin A treatment

For the Brefeldin A (BFA, Sigma-Aldrich) treatment *D*. *flagrans* was co-incubated with a mixed population of *C*. *elegans* N2 on LNA slides for 24 h at 28°C. Next, a 5 μl drop of a 50 μg/ml BFA solution was placed on a glass slide and a 0.5 x 0.5 cm square of the LNA slide with the induced fungus was placed up-side-down onto the drop. Localization of CyrA-mCherry was then observed under the fluorescent microscope.

### Virulence assay

For the long-term observation spores were incubated on LNA microscopy slides for 24 h together with a mixed population of *C*. *elegans* N2. The worms were washed off the slides afterwards and synchronized L3 or L4 larvae of the strain BAN126 were added to the now induced mycelium. Empty trapping networks were observed with a Zeiss AxioObserver Z1 inverted microscope and pictures were taken in the brightfield and GFP channel every 2 minutes for 12 hours.

### Lifespan assay

For the lifespan assay 30 L4 stage nematodes were transferred to NGM plates supplemented with 150 μM 5-fluoro-2′-deoxyuridine (FUdR). The plates were cultured at 20°C and the number of live and dead nematodes was determined with a dissecting microscope every day. The assay was conducted with at least 100 worms in two independent experiments. Survival rates were calculated using the Kaplan-Meier method and survival rates were evaluated using the log-rank test (P<0.05) using OASIS (https://doi.org/10.18632/oncotarget.11269)

### Statistical analyses

P-values were calculated with GraphPad Prism applying the student’s t-test. Experiments were conducted in three technical and biological replicates.

### Microscopy

For microscopical analyses 4x10^4^ spores were inoculated on thin LNA slides and incubated for at least 12 h at 28°C. Conventional fluorescence images were captured at room temperature using a Zeiss Plan-Apochromat 63x/1.4 Oil DIC, EC Plan-Neofluar 40x/0.75, EC Plan-Neofluar 20x/0.50, or EC Plan-Neofluar 10x/0.30 objective with a Zeiss AxioImager Z.1 and AxioCamMR. For Confocal microscopy, the Zeiss LSM 900 with Airyscan 2 was used. Images were collected using ZEN 2012 Blue Edition.

Stereomicroscopy was performed using a Zeiss Lumar.V12 with AxioCam HRc and NeoLumar S 1.5x objective. Images were collected with the AxioVision software.

For the long-term observation virulence assay of *D*. *flagrans* wild-type and the Δ*cyrA* strain an AxioObserver Z1 inverted microscope employing a 10x/0.30 N.A. objective (Zeiss) was used.

The fungal cell wall was visualized by Calcofluor-white (CFW, fluorescent brightener 28, Sigma Aldrich) as described [15].

## Supporting information

S1 Movie Accumulation of CyrA-GFP at the infection bulb during the attack. The CyrA-GFP expressing strain sNH30 was co-incubated with *C*. *elegans* on thin LNA slides for 24 h at 28°C. Pictures of *C*. *elegans* captured by *D*. *flagrans* were taken in brightfield and the GFP channel every minute and assembled into a movie sequence. The fusion protein emerges from three penetration sites in the head region of the nematode at minute 42.
(MP4)

S2 Movie Brefeldin A treatment inhibits penetration of *C*. *elegans*. The *D*. *flagrans* strain sNH65 expressing *cyrA(p)*::*cyrA*::*mCherry* was co-incubated with *C*. *elegans* on thin LNA slides for 24 h at 28°C. A 1 cm square was cut from the agar and placed upside down onto a 5 μl drop of 50 μg/ml BFA. Pictures of captured worms were taken every 5 minutes and assembled into a movie sequence. Three worms are captured by a trapping network. No entry site, bulb formation or CyrA accumulation is visible.
(MP4)

S3 Movie Accumulation of CyrA-mCherry at the infection bulb under conditions without any treatment. The *D*. *flagrans* strain sNH65 expressing *cyrA(p)*::*cyrA*::*mCherry* was co-incubated with *C*. *elegans* on a thin LNA slide for 24 h at 28°C. A 1 cm square was cut from the agar and placed upside down onto a drop of water. The CyrA-mCherry signal emerges in the head region after 35 min. The accumulation at the infection bulb is clearly visible after 60 min.
(MP4)

S4 Movie The exocyst component *exoA* is necessary for CyrA accumulation at the infection bulb. The *ΔexoA-*mutant strain (sNH60) expressing CyrA-mCherry was co-incubated with *C*. *elegans* on thin LNA slides for 24 h at 28°C. CyrA accumulates at the infection site on the lower left side of the trapped nematode after 10 minutes. The infection bulb is visible after 30 minutes, but CyrA does not accumulate in the bulb, instead some signal is visible at the outer rim of the entrance site while the trophic hyphae stretch throughout the nematode body. After 60 min the nematode is partly colonized but the fusion protein is not visible inside of the worm.
(MP4)
